# Evidence of salt accumulation in beach intertidal zone due to evaporation

**DOI:** 10.1038/srep31486

**Published:** 2016-08-11

**Authors:** Xiaolong Geng, Michel C. Boufadel, Nancy L. Jackson

**Affiliations:** 1 Center for Natural Resources Development and Protection, Department of Civil and Environmental Engineering, New Jersey Institute of Technology, Newark, NJ 07102, United States; 2 Department of Chemistry and Environmental Science, New Jersey Institute of Technology, Newark, NJ 07102, United States

## Abstract

In coastal environments, evaporation is an important driver of subsurface salinity gradients in marsh systems. However, it has not been addressed in the intertidal zone of sandy beaches. Here, we used field data on an estuarine beach foreshore with numerical simulations to show that evaporation causes upper intertidal zone pore-water salinity to be double that of seawater. We found the increase in pore-water salinity mainly depends on air temperature and relative humidity, and tide and wave actions dilute a fraction of the high salinity plume, resulting in a complex process. This is in contrast to previous studies that consider seawater as the most saline source to a coastal aquifer system, thereby concluding that seawater infiltration always increases pore-water salinity by seawater-groundwater mixing dynamics. Our results demonstrate the combined effects of evaporation and tide and waves on subsurface salinity distribution on a beach face. We anticipate our quantitative investigation will shed light on the studies of salt-affected biological activities in the intertidal zone. It also impacts our understanding of the impact of global warming; in particular, the increase in temperature does not only shift the saltwater landward, but creates a different salinity distribution that would have implications on intertidal biological zonation.

The salinity structure in beaches subjected to tides and waves involves complex behavior due to seawater-groundwater circulation and mixing^1^,^2^,^3^,^4^,^5^, which affect beach bio-geochemical processes, such as nutrients transformation^6^,^7^,^8^ and ecological functions^9^,^10^,^11^,^12^. It is commonly assumed that the high salinity observed in the intertidal zone of a beach is that of seawater^4^,^13^,^14^, which is due to neglecting the evaporation from the beach surface. We report herein recently acquired field results in Delaware Bay, USA (Fig. 1), where the pore water salinity in the intertidal zone was much larger than that of seawater. We also interpret the results using numerical modeling.

The studied beach is an eroding sandy former overwash barrier modified by artificial beach nourishment and construction of a dune dike 2.3 m above backshore elevation (Figure S1). Beach sediments are moderately to moderately-well sorted medium to coarse sands (sediment size D_10_ ranges from 260.1 μm to 740.4 μm). We measured groundwater table using pressure transducers installed in four well points (PW1–PW4) across the beach foreshore. We also measured pore-water salinity at four depths by coring sediment samples almost every 2 h over complete tidal cycles at four locations adjacent to the wells for 7 discontinuous days over a month. Beach topography was surveyed during the study period (Figure S1). Local climatological data were obtained from NOAA’s National Climatic Data Center (Figure S2).

Figure 2 shows the measured pore-water salinity at PW1 was uniform within 20 cm depth at 7 am. However, it increased throughout the depth at 9:30 AM with the top 5 cm layer’s salinity separating in value from the rest. The following measurement at noon showed an even greater separation, where the top 5 cm layer salinity increased to 70 ± 7 g/L while the remainder of the column had a salinity close to that of seawater. The salinity in the top layer at PW2 was close to that of seawater at 7:00 and 9:30 AM, around 26 ± 3 g/l, and increased at noon to 45 ± 5 g/l at 2:00 PM (on the falling tide). The salinity at deeper locations of PW2 increased only slightly with time. The salinity at PW3 and PW4 was more or less uniform with depth and constant (with time). Figure 3a shows that on June 9^th^, the pore-water salinity at top 5 cm layer at PW1 significantly increased from 24 ± 2 g/l to 160 ± 16 g/l, followed by a rapid decrease to 77 ± 8 g/l, during rising tide. The pore-water salinity increased again (up to 220 ± 22 g/l) after the groundwater table reached its peak and started to drop at subsequent falling tide. A similar behavior was noted during other sampling days where the increase in salinity tended to be attenuated when groundwater was close to the beach face, for example, on June 4^th^ at PW1–PW4 (Figure S5) and on June 16^th^ and 18^th^ at PW2 and PW3 (Figures S7 and S8). Figure 3b reports all the observed pore-water salinity at the top 5 cm layer along with the temporal average, which took the values 56 g/l, 36 g/l, 32 g/l, and 30 g/l at PW1, PW2, PW3, and PW4, respectively. Thus, even at seaward locations, the average salinity was consistently larger than seawater (27 ± 3 g/l). It is also important to note that the pore water salinity exceeded 50 g/l and 40 g/l at PW2 and PW3, respectively. The variability of the pore-water salinity decreased going seaward.

Our results (Figs 2 and S3–S8) suggest that the measured subsurface intertidal salinity, especially in the top beach layers, correlated strongly with the diurnal cycle. In the morning, humid atmospheric conditions resulted in negligible or no evaporation from the beach. During daylight, especially around noon, the relatively high air temperature and low humidity caused high evaporation, extracting pore water from the beach and leaving the salt behind, thereby resulting in high salinity near the beach surface. The evaporation caused dramatic salinity zonation within the intertidal zone; at the upper intertidal zone, pore-water salinity near the beach surface significantly increased around noon, but the increase was smaller in the mid-intertidal zone, most likely due to more frequent inundation by tide, which not only diluted the pore water, but also provided sufficient new water so that the evaporation no longer affected the existing pore water. The observed swash limit indicates that the pore-water flow and salinity structure in the beach was also impacted by waves. Similar to tidal action, high-frequency waves drove seawater to infiltrate through the beach swash zone^15^,^16^,^17^ and thereby attenuated evaporation-induced salinity increase there.

The pore-water salinity near the beach surface was also dependent on beach capillarity, namely on water retention. For example, if evaporation removes 10% of the pore volume (of the porosity), then a decrease in moisture from 80% to 70% would increase the salinity by 11%, but a decrease from 15% to 5% increases the salinity by 300%. For this beach, the sand cores were used to obtain capillary-retention properties, and along with calibration^18^, the capillary fringe was found to be approximately between 0.2 m and 0.5 m. As the groundwater table rose, the capillary fringe provided water to the beach surface from below. This could result in two opposing outcomes: when meteorological conditions favor high evaporation, the capillary fringe would result in higher pore-water near the beach surface, which would subsequently evaporate and increase the salinity, as reported by Geng and Boufadel^19^ for inland environments. But under unfavorable meteorological conditions for evaporation, the rise of water by capillarity would dilute existing high pore-water salinity at the beach surface.

Our findings indicate that saturated-flow models (e.g., MODFLOW) would not capture the increase in pore water salinity, which occurred herein under unsaturated-flow conditions. For this reason, we used the density-dependent variably saturated model MARUN^20^ to conduct simulations to interpret the May 19^th^ results (Fig. 2). The simulations were conducted based on the conceptual/numerical approach in Figure S9, and the results shown in Figure S10 captured in general the observed pore-water salinity.

Figure 3c shows simulated salinity contours along with moisture content in the beach at 11:30 AM on May 19^th^. The pore-water salinity at the beach surface at *x* = 10 m was around 100 g/l. The sediment moisture dropped gradually from 99% to 75% (15% drop over 0.20 m) and then sharply until 20% (55% drop over 0.20 m), which is consistent with a capillary fringe of around 0.40 m based on data and calibration (Table S1). The velocity vectors clearly show upwelling of water flow in the unsaturated zone, which is due to evaporation. Our simulation results indicate that evaporation-induced hypersaline conditions seemed to appear within top 5 cm layer of the beach where water saturation was less than 20% when this beach segment was exposed to atmosphere. Due to the low moisture there, the inundation during the subsequent tidal cycles is likely to dilute the salt concentration significantly. Therefore, in presence of evaporation, the near-surface pore-water salinity tends to increase during low tides and drop to seawater salinity at high tides due to inundation.

We used the observed meteorological condition and tide level on May 19^th^ (Figure S11a,b) as a base case to conduct a sensitivity analysis, and we altered each according to Table S2 to numerically evaluate the effects of meteorological conditions on beach hydrodynamics. Figure S12 shows that the moisture ratio at the top layer of PW1 was generally smaller than 10%, while that at PW2 reached 100% at high tide, even when the water table was below the beach surface. This is due to the fill-up of the beach from below due to capillary rise.

Figures S13 shows that the evaporation rate was relatively large at PW3 and PW4, but the maximum values were noted at PW2. PW1 exhibited the lowest evaporation rate, which is due to low moisture at that location (Figure S12). This indicates that evaluating the evaporation rate on the beach surface might not be sufficient to explain the salinity distribution, and the converse is also true. Therefore, the combination of surface and subsurface monitoring is essential for delineating intertidal subsurface hydraulic exchange and pore-water salinity structure.

Figures S14–16 show simulated salinity distribution in the top layer of the beach at low and high tides under different meteorological condition. It is shown that increasing the temperature, decreasing the relative air humidity, and/or increasing wind speed increased the pore-water salinity at PW2 and landward locations. Temperature and relative humidity appeared to have more drastic effects on pore-water salinity, especially during low tides. Figures S17–19 elucidate the nonlinear temporal variation of salinity. By contrast to the spatial distribution, the temporal variation (at PW1) seemed to be affected the most by the relative humidity that caused wild temporal variation in pore-water salinity.

In inland environments, evaporation would increase the pore water concentration more or less uniformly across vast distances^19^,^21^. However, this study showed that the salinity in the pore of an intertidal beach could vary over a large range within only a few hours and within a short distance, and it could reach an extremely high value (up to 200 g/l) that only halophilic bacteria can tolerate^22^,^23^. Our numerical investigation revealed that an increase in temperature or a decrease in relative humidity (e.g., due to climate change) would not only increase the pore-water salinity in the beach, but would also alter its spatial distribution; abrupt salinity increases are expected to occur immediately near the water line. This change in salinity zonation has consequences on biogeochemical reactions in pore water and on the ecological function and structure of beaches^24^,^25^,^26^,^27^. This study was conducted in the intertidal zone of the beach. In the supratidal zone of beach, evaporation could persist for longer time and salt-feeding would take place due to large wave run-up or unsaturated flow of ambient saline water. Therefore, evaporation might demonstrate more remarkable effects on supratidal pore-water salinity. Field campaign and numerical modeling need to be carried out in beach supratidal zone to further examine the complex interaction between surface evaporation and subsurface saline pore-water flow such as the formation of surficial salt crust and the balance of salt structure affected by multiple surface and subsurface factors. In addition, the slope of our studied beach is approximately 0.1, which is much steeper than many natural beaches. Beach slope might affect the extent of intertidal zone across the shoreline and subsequently alter exposure time of the beach segment to evaporation. Therefore, topography could be another factor affecting subsurface salt distribution in coastal beaches subjected to evaporation, tides and waves, which needs to be considered in future.

## Methods

### Field Measurements

Groundwater table was measured using pressure transducers (Druck PDCR1830) placed in four 41.0 mm internal diameter commercial well points installed from the break in slope to the spring swash limit. Water level was measured from a pressure transducer placed 35 m seaward of the break in slope. Data from these instruments were recorded every second and average over 5 min intervals. Data to determine wave height and period were collected near times of daytime high water using a pressure transducer placed on the lower of foreshore. These wave data were recorded at 8 Hz in 17.1 min bursts. Beach elevation changes were monitored at 2 m intervals along a cross-shore transect during each daytime low tide.

### Pore-water Salinity Measurements

Pore-water salinity was determined from core samples, 0.2 long and 0.05 m in diameter, gathered across the intertidal foreshore at four well locations. Core samples were partitioned into four 0.05 m slices. Sediments were weighted, air-dried and reweighed to determine gravimetric water content. Each air-dried sediment sample was put into a flask with 70 ml deionized water, and then shaken for 12 hours to make the salt dissolve completely in the water. Salinity was measured directly from 25 ml water filtrated from the sediment solution to determine the total salt mass in the sediment samples. The pore-water salinity was calculated as the ratio of the salt mass to the mass of pore water.

### Numerical Simulations

Numerical simulations were conducted using the MARUN (MARine Unsaturated) model^20^, which can simulate two-dimensional density-dependent flow and solute transport in variably saturated porous media. The variable pore-water saturation, relative permeability and capillary pressure were described by the van Genuchten model^28^. The evaporation rate from the beach was estimated using the bulk aerodynamic approach^29^, which was coupled with the MARUN model^30^. The resulting evaporation rate became thus dependent on both the atmospheric and subsurface dynamics.

## Additional Information

**How to cite this article**: Geng, X. *et al*. Evidence of salt accumulation in beach intertidal zone due to evaporation. *Sci. Rep.* **6**, 31486; doi: 10.1038/srep31486 (2016).

## Supplementary Material

Supplementary Information

## Figures and Tables

**Figure 1:**
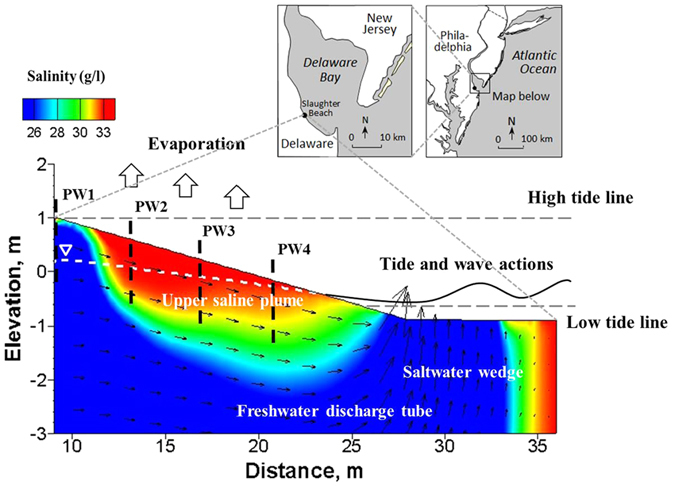
Location of the study site and topography of the beach transect. Four piezometer wells (PW1−PW4) were installed along the intertidal zone of the beach to monitor groundwater table fluctuation due to tidal action. The mean sea level was assigned as the elevation datum (0.0 m). Major processes of subsurface pore water flow and salt fate are illustrated in the Figure, including the upper saline plume, the freshwater discharge tube, the classic saltwater wedge, and pore water evaporation from the beach surface. Note the exaggerated vertical scale. The map of the studied site is obtained from Jackson *et al*.^31^.

**Figure 2:**
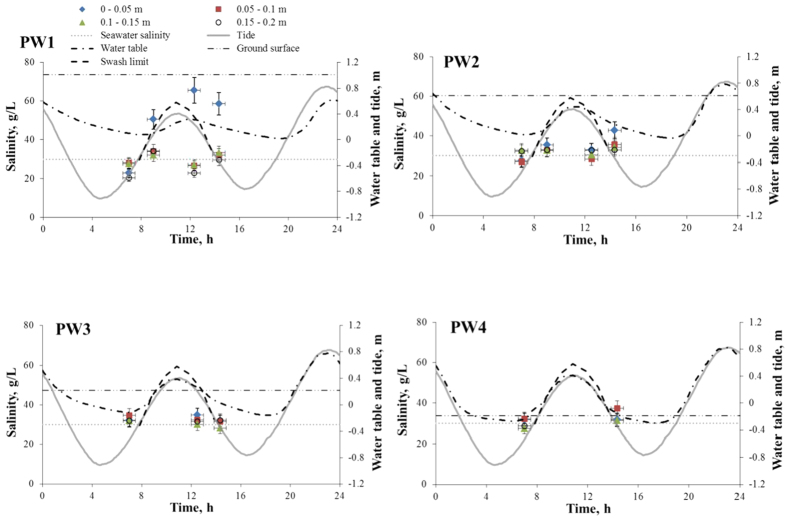
Pore-water salinity measured during May 19^th^ at four depths (0–0.05 m, 0.05–0.1 m, 0.1 m–0.15 m, and 0.15 m–0.2 m) at four locations (PW1–PW4) along with the measurements of tide and groundwater table. High pore-water salinity was observed at PW1 and PW2 at the top 5 cm of the beach. Time 12 is noon.

**Figure 3:**
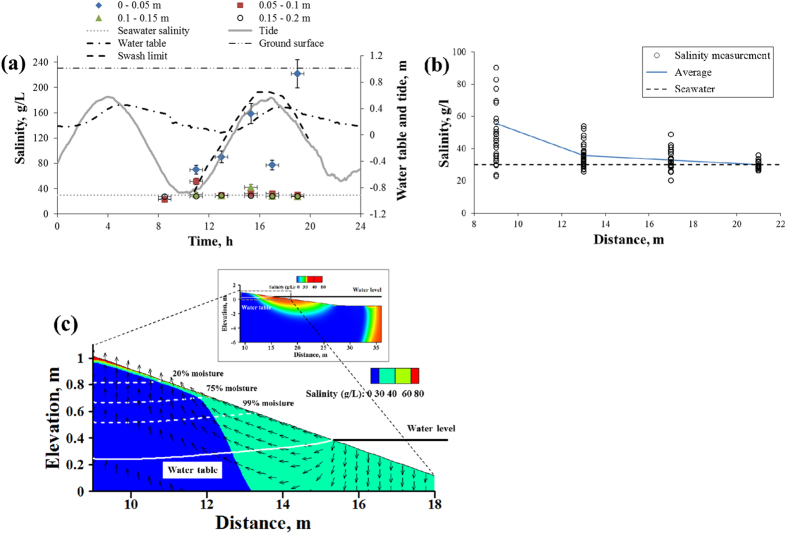
(**a**) Pore-water salinity measured during June 9^th^ at four depths (0–0.05 m, 0.05–0.1 m, 0.1 m–0.15 m, and 0.15 m–0.2 m) at PW1 location along with the measurements of tide and groundwater table. (**b**) Observed salinity profile at the top 5 cm layer of the beach at different times. Two values 160 g/l and 220 g/l are not reported in the figure for visual clarity. They contributed to the average value. (**c**) Simulated salinity contour at 11:30 AM on May 19^th^. White dashed lines denote moisture content (percentage by volume) and the white solid line denotes groundwater table. The velocity vectors indicate only the pore-water velocity direction, but not its magnitude. Also, note that due to the exaggeration of the vertical scale, the velocity vectors do not appear perpendicular to the beach surface (as the beach slope was visually increased). Extremely high pore-water salinity was measured at PW1 at the top 5 cm; in contrast, pore-water salinity at other depths and/or locations was almost the same as seawater (shown in Figure S6). The simulated contour indicates that a high saline layer was formed along the beach surface due to evaporation.
