# Author Correction: Evidence of salt accumulation in beach intertidal zone due to evaporation

**DOI:** 10.1038/s41598-020-62763-9

**Published:** 2020-04-02

**Authors:** Xiaolong Geng, Michel C. Boufadel, Nancy L. Jackson

**Affiliations:** 1 Department of Civil and Environmental Engineering, Center for Natural Resources Development and Protection, New Jersey Institute of Technology, Newark, NJ 07102 United States of America; 2 Department of Chemistry and Environmental Science, New Jersey Institute of Technology, Newark, NJ 07102 United States of America

Correction to: *Scientific Reports* 10.1038/srep31486, published online 11 August 2016

The Acknowledgements section in this Article is incomplete.

“This research paper was made possible in part by a grant from The Gulf of Mexico Research Initiative to the Consortium CARTHE II, and subsequently to the New Jersey Institute of Technology. Funding has also come from the National Science Foundation under grant CBET-1313185 and New Jersey Sea Grant with funds from NOAA Office of Sea Grant, US Department of Commerce, under NOAA grant # NA10OAR4170075. NJSG-16-905. However, no official endorsement should be implied from any of the funding entities.”

should read:

“This research paper was made possible in part by a grant from The Gulf of Mexico Research Initiative to the Consortium CARTHE II, and subsequently to the New Jersey Institute of Technology. Funding has also come from the National Science Foundation under grant CBET-1313185 and New Jersey Sea Grant with funds from NOAA Office of Sea Grant, US Department of Commerce, under NOAA grant # NA10OAR4170075. NJSG-16-905. Data are publicly available through the Gulf of Mexico Research Initiative Information and Data Cooperative (GRIIDC) at https://data.gulfresearchinitiative.org (DOI: 10.7266/N7W9577H). However, no official endorsement should be implied from any of the funding entities.”

