# Supplementary material for: Evidence of salt accumulation in beach intertidal zone due to evaporation
Source: Sci Rep. 2016 Aug 11;6:31486. doi: 10.1038/srep31486 (PMC4980607; doi:10.1038/srep31486)
Supplement: Supplementary Information [file srep31486-s1.pdf]

Evidence of salt accumulation in beach intertidal zone due to evaporation

Xiaolong Geng[1], Michel C. Boufadel[1*], and Nancy Jackson[2]

1. Center for Natural Resources Development and Protection,
Department of Civil and Environmental Engineering, New Jersey Institute of Technology, Newark, NJ 07102, United States

2. Department of Chemistry and Environmental Science, New Jersey Institute of Technology, Newark, NJ 07102, United States

*: Corresponding author. boufadel@gmail.com, Center for Natural Resources Development and Protection, New Jersey Institute of Technology, Newark, NJ 07102, United States

Supplementary Material

This supplementary provides additional data from the field site at Slaughter beach, Delaware, along with further numerical modeling results. The material is organized as two tables and nineteen figures: nine for field measurements (Supplementary Figures 1-8 and 11) and twelve for the numerical modeling (Supplementary Tables 1 and 2, and Supplementary Figures 9-10 and 12-19).

Field results are shown in Supplementary Figures 1-8 including beach topography survey (Supplementary Figure 1), meteorological conditions (temperature and relative humidity shown in Supplementary Figure 2), and measurements of pore-water salinity, groundwater table, and tides (Supplementary Figures 3-8). The measurements of tide, groundwater table, temperature, and relative humidity in the air in the selected period for sensitivity analysis are shown in Supplementary Figure 11.

The simulated domain is shown in Supplementary Figure 9. The parameter values used for the simulation and the thirteen sets of meteorological conditions used for the sensitivity analysis are listed in Supplementary Table 1 and 2, respectively. Simulation results conducted are shown in Supplementary Figures 10 and 12-19, including beach shallow layer pore-water salinity (Supplementary Figure 10), moisture ratio (Supplementary Figure 12), and evaporation rate (Supplementary Figure 13). Results of thirteen simulations are summarized in Supplementary Figures 14-19 to illustrate model sensitivity to air temperature, relative humidity, and wind speed. Effects of air temperature, relative humidity, and wind speed on spatial distribution of pore-water

salinity at top 5 cm layer along beach transect are shown in Supplementary Figures 14-16, respectively. Effects of temperature, relative humidity, and wind speed on temporal change of pore-water salinity at the PW1 location are shown in Supplementary Figures 17-19.

Supplementary Table 1: Model parameter values used in the numerical simulations
The number of nodes was 12,358 nodes (74 nodes in horizontal direction and 167 nodes in the vertical direction). Details on the spacing are reported in Supplementary Figure 9

Symbol	Definition	Units	Value
α	Parameter of the van Genuchten [1980] model	m^{-1}	2.0^{*}
n	Parameter of the van Genuchten [1980] model	-	8.1^{*}
K_0	Saturated freshwater hydraulic conductivity	ms^{-1}	$4.73\times10^{-4**}$
α_L	Longitudinal dispersivity	M	0.02
α_T	Transverse dispersivity	m	0.002
ξ	Slope of the density concentration relationship	lg^{-1}	6.46×10^{-4}
S_0	Specific storage	m^{-1}	10^{-5}
S_r	Residual soil saturation	-	$5.0\times10^{-3*}$
Φ	Porosity	-	0.3
CONVP	The convergence criterion of pressure head in the Picard iterative scheme of MARUN code	m	10^{-5}
τD_m	Product of tortuosity and diffusion coefficient	m^2s^{-1}	10^{-10}

* The capillary parameter values estimated by capillary retention experiments were α = 4.5 m^{-1} and n = 8.1. These values were used in a conceptual Bayesian approach as prior estimates for the calibrated values of α = 2.0 m^{-1} to match what was observed for salinity in the field.
** Saturated freshwater hydraulic conductivity was estimated based on the grain size analysis using the Kozeny-Carman equation[1].

Supplementary Table 2. Atmospheric conditions for the numerical experiments conducted.

Case	Temperature (^oC)	Relative humidity (-)	Wind speed (m/s)
1	Observation*	Observation	2.0
2	Observation – 10 ^oC	Observation	2.0
3	Observation + 10 ^oC	Observation	2.0
4	Observation + 15 ^oC	Observation	2.0
5	Observation	Observation + 5%	2.0
6	Observation	Observation – 5%	2.0
7	Observation	Observation – 10%	2.0
8	Observation	Observation – 15%	2.0
9	Observation + 10 ^oC	Observation – 5%	2.0
10	Observation	Observation	1.0
11	Observation	Observation	4.0
12	Observation	Observation	6.0
13	No evaporation		

* Temperature and relative humidity were obtained from NOAA, National Climatic Data Center with an interval of 3 hours shown in Figures S2.

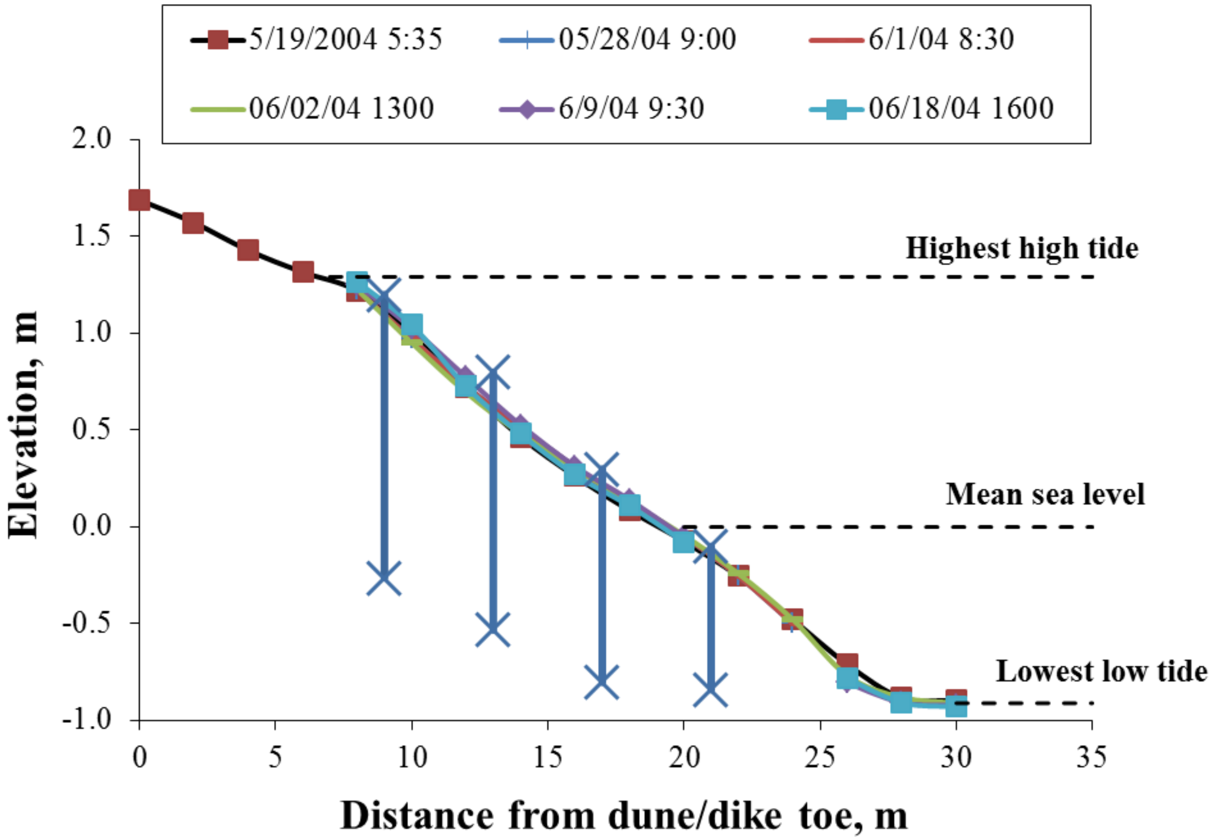

Supplementary Figure 1: Topography of the beach transect surveyed at six different days during the investigated period. The mean sea level was assigned the elevation datum (0.0 m). Frequent topographic surveys revealed that no significant change in beach topography occurred during the investigated period.

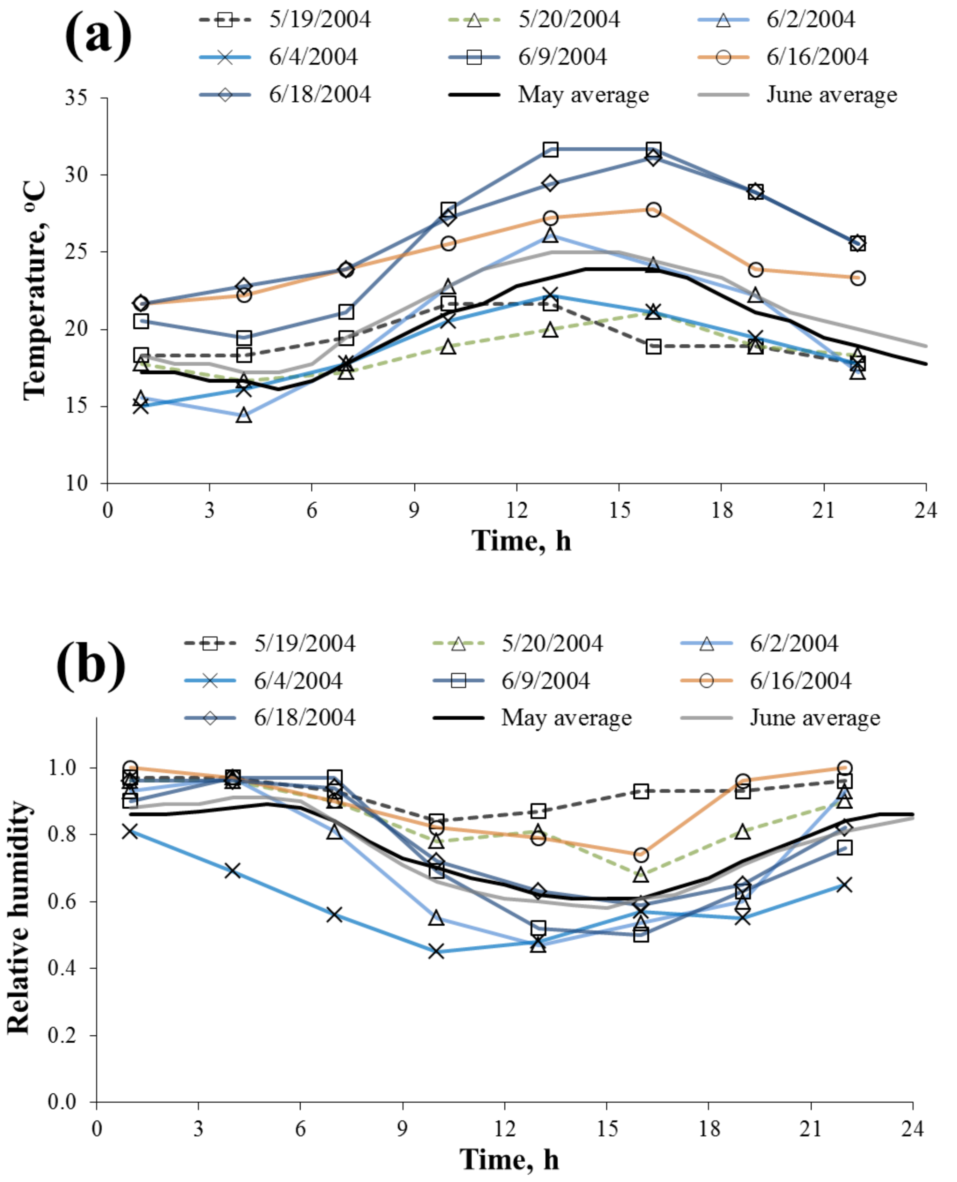

Supplementary Figure 2: measurements of (a) relative humidity and (b) temperature. The data were collected from NOAA, National Climate Data Center with an interval of 3 hours, around 50 miles from the beach. The data show that the temperature fluctuated within the 24 hour period, reaching a maximum at noon and dropping to minima at midnight; in general, the observed temperature in May and June is, respectively, slightly lower than and much high than the monthly average. The relative humidity in the air was comparatively low in the day and high during the night. The data of relative humidity from the observed periods are representative, as the values are similar to (June 18th), or higher than (May 19th, May 20th, and June 16th), or lower than (June 4th and June 9th) the monthly average. No precipitation occurred during the study period.

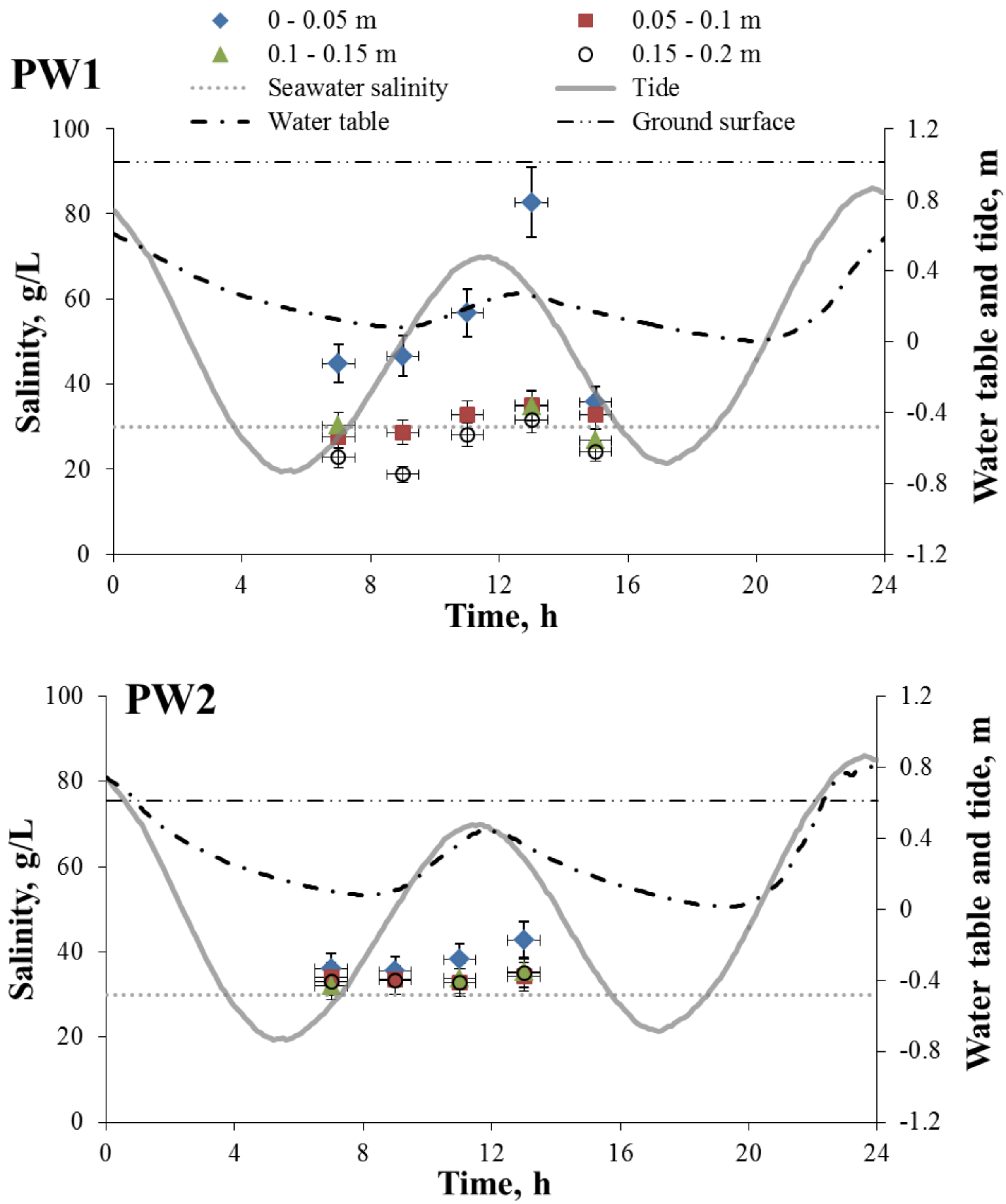
0 - 0.05 m
0.05 - 0.1 m
0.1 - 0.15 m
0.15 - 0.2 m
Seawater salinity
Tide
Water table
Ground surface
PW1
Salinity, g/L
Water table and tide, m
Time, h
PW2
Salinity, g/L
Water table and tide, m
Time, h

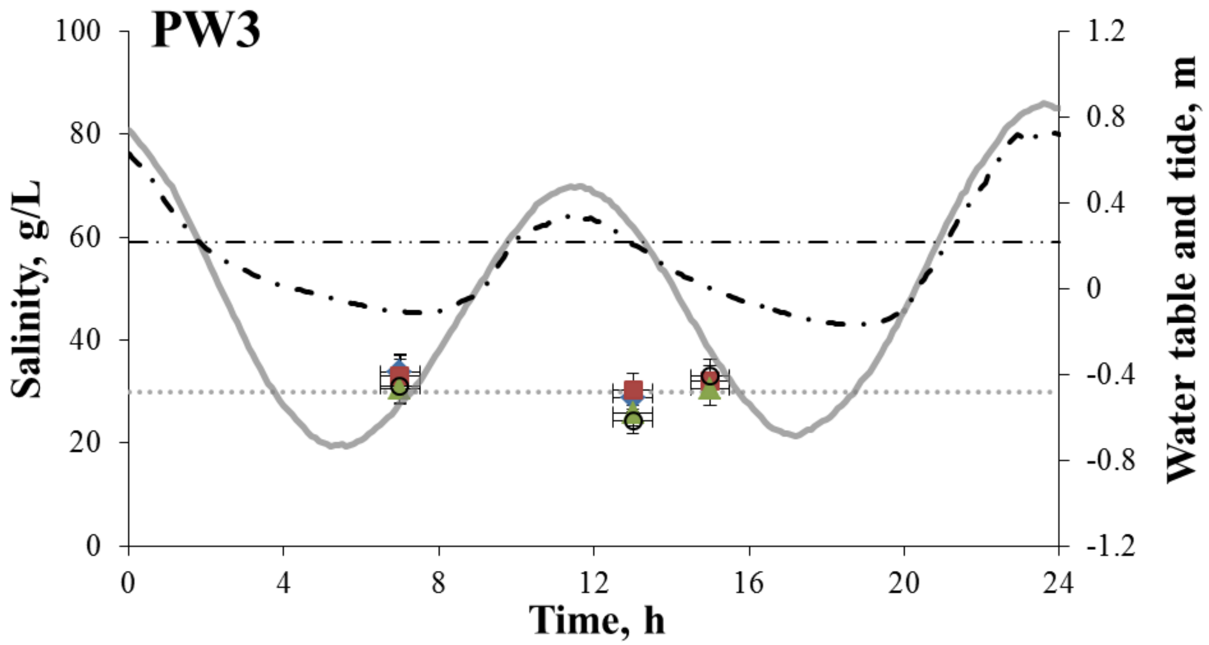

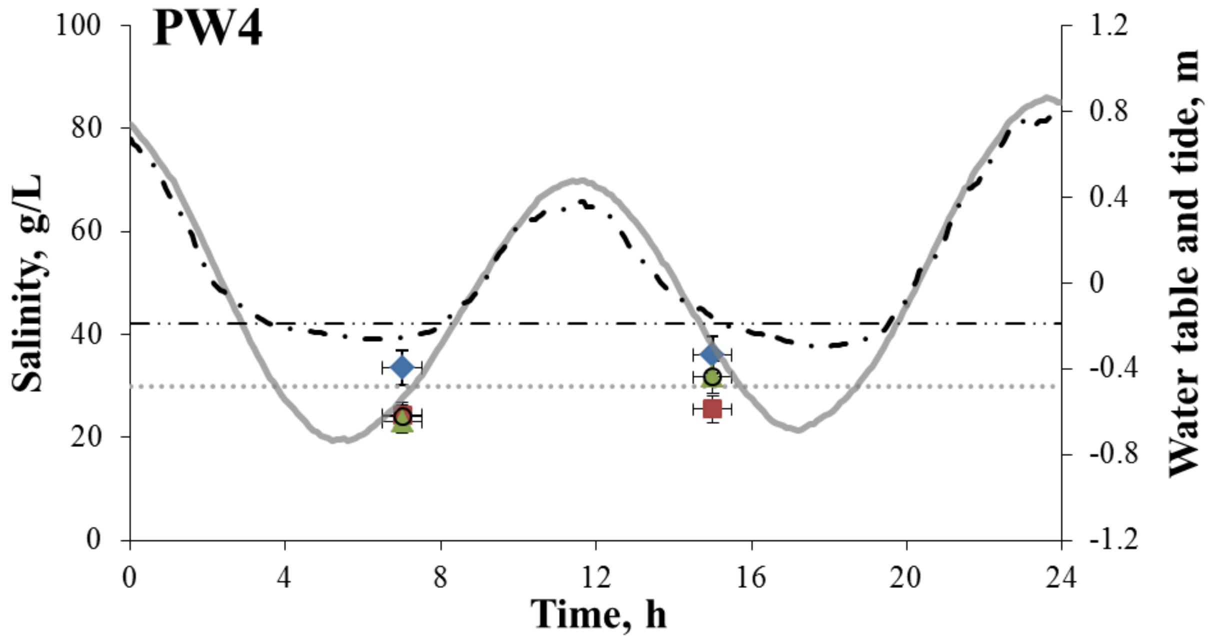

Supplementary Figure 3: Measured pore-water salinity during May 20th at four locations (PW1-PW4, Figure 1) and four depths, along with measurements of tide and groundwater table. Notice that high pore-water salinity was observed at the beach top layer at the landward wells PW1 and PW2.

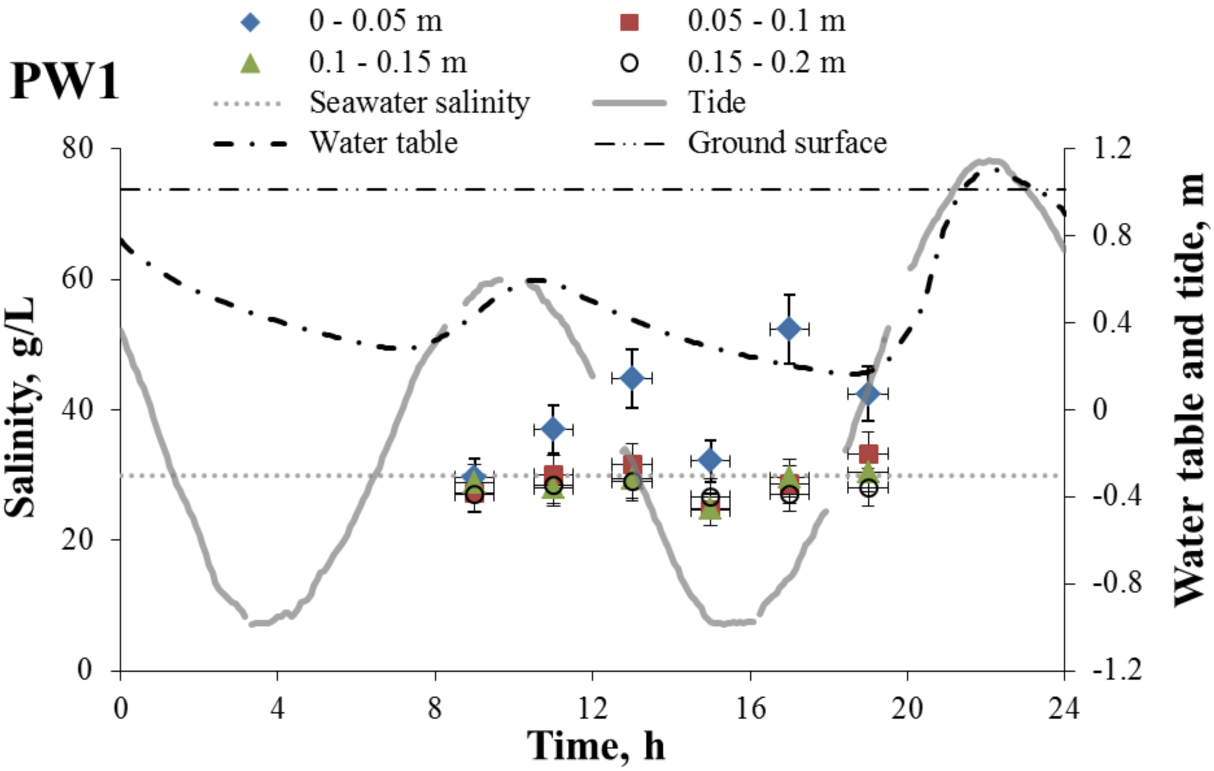

PW1
0 - 0.05 m
0.05 - 0.1 m
0.1 - 0.15 m
0.15 - 0.2 m
Seawater salinity
Tide
Water table
Ground surface
Salinity, g/L
Water table and tide, m
Time, h
0
4
8
12
16
20
24
20
40
60
80
1.2
0.8
0.4
-0.4
-0.8
-1.2

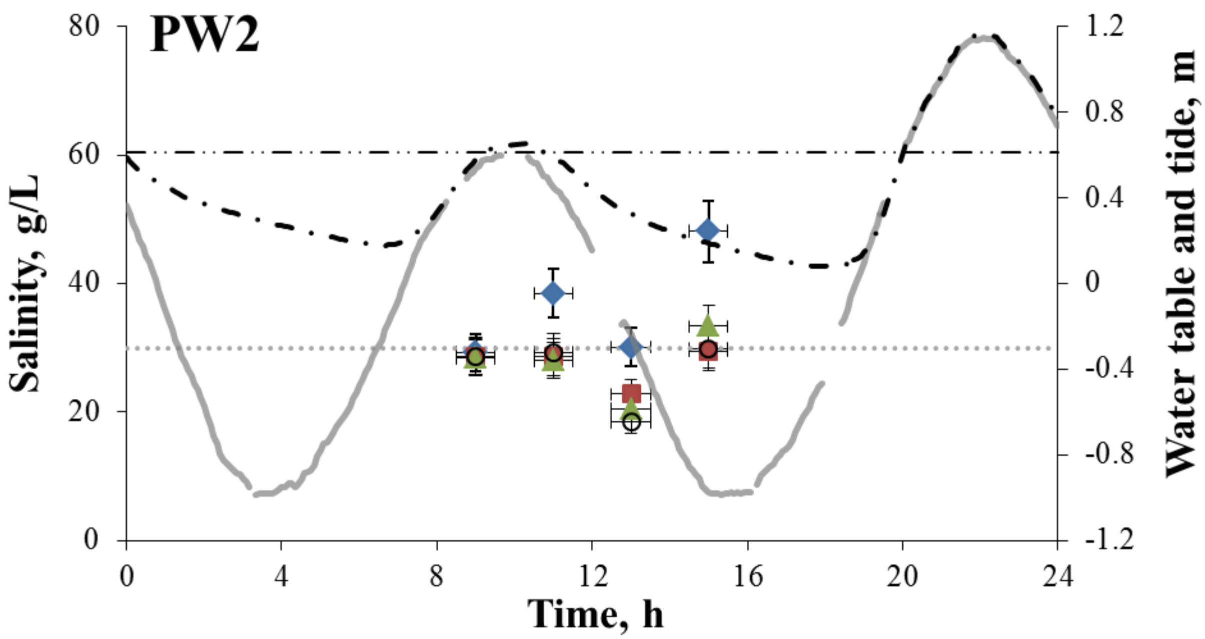
PW2
Salinity, g/L
Water table and tide, m
Time, h
0
20
40
60
80
1.2
0.8
0.4
0
-0.4
-0.8
-1.2
0
4
8
12
16
20
24

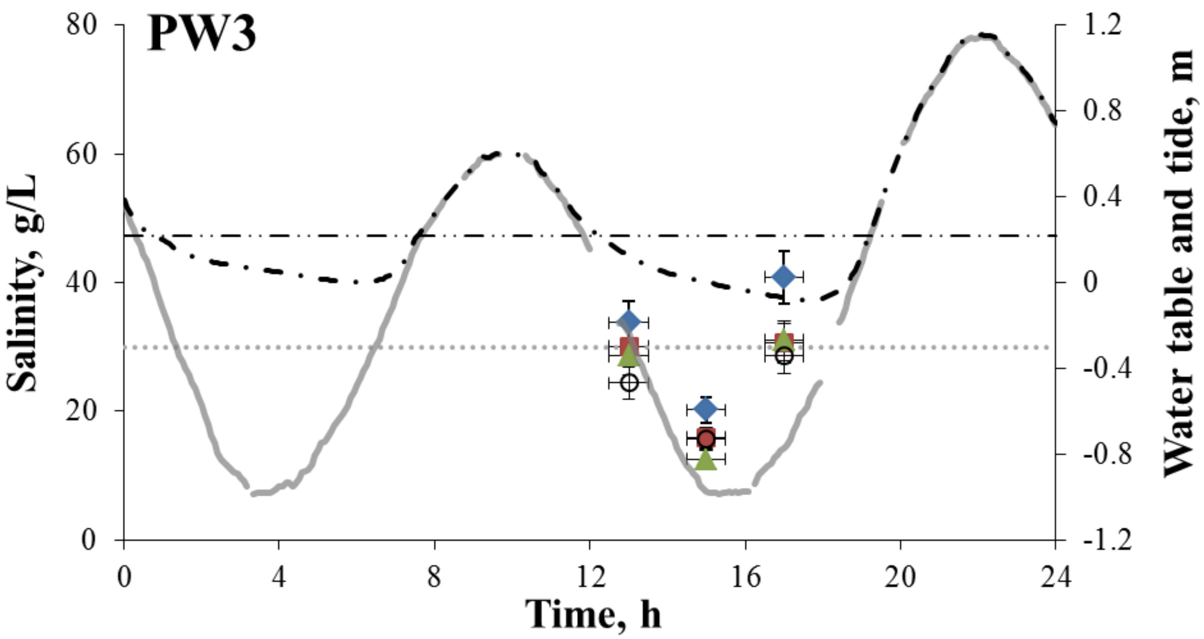
PW3
Salinity, g/L
Water table and tide, m
Time, h
0
20
40
60
80
1.2
0.8
0.4
0
-0.4
-0.8
-1.2
0
4
8
12
16
20
24

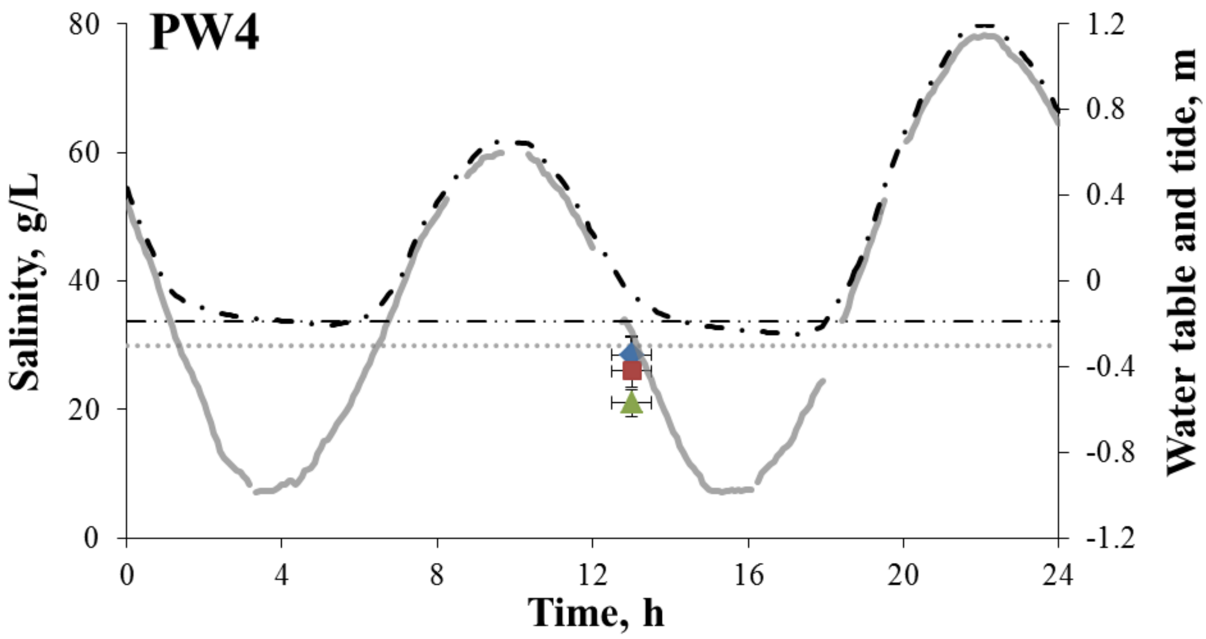

Supplementary Figure 4: Measured pore-water salinity during June 2nd at four locations (PW1-PW4, Figure 1) and four depths, along with measurements of tide and groundwater table. Notice that high pore-water salinity was observed at the beach top layer at the landward wells PW1 and PW2.

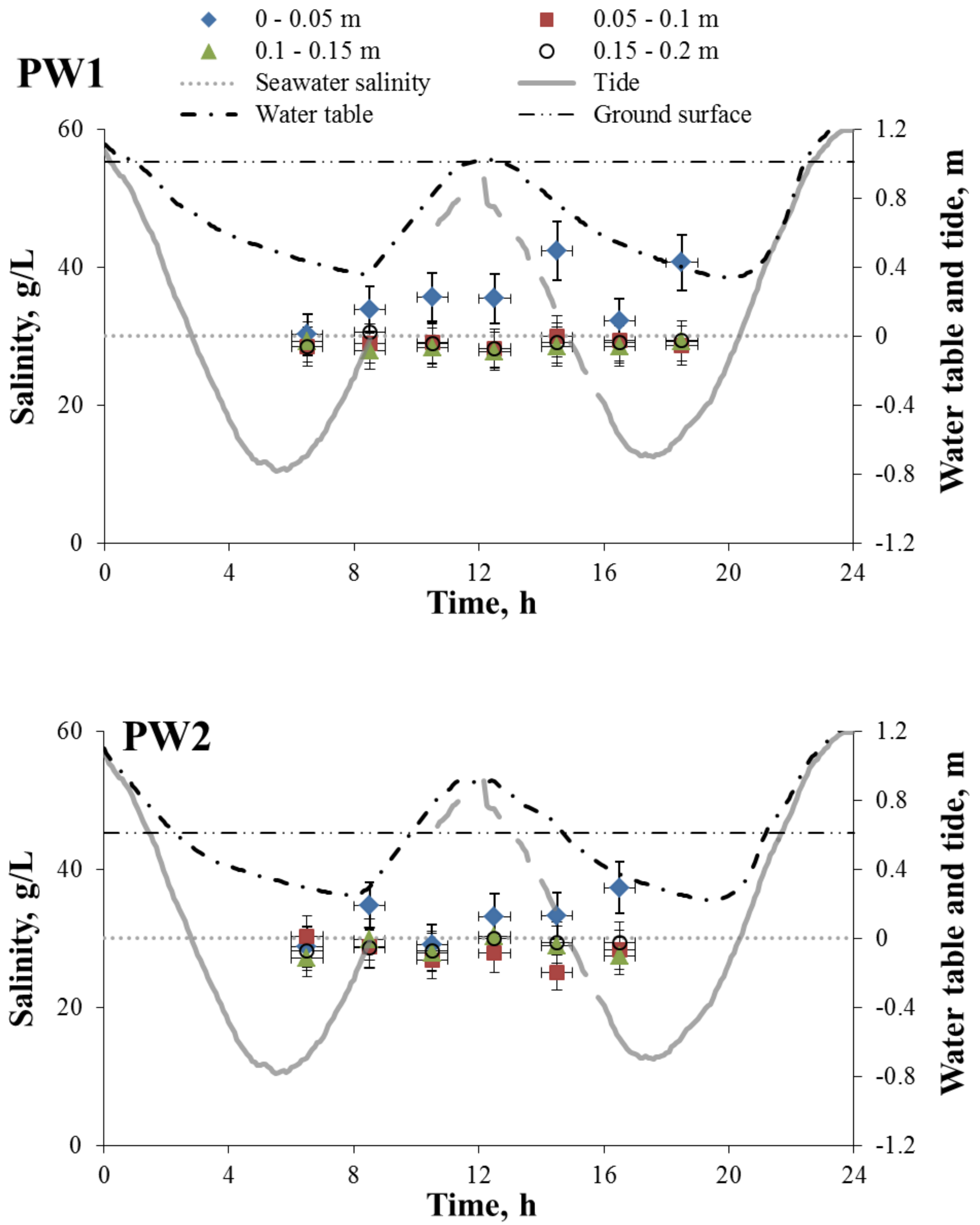

0 - 0.05 m
0.05 - 0.1 m
0.1 - 0.15 m
0.15 - 0.2 m
Seawater salinity
Tide
Water table
Ground surface
PW1
Salinity, g/L
Water table and tide, m
Time, h
PW2
Salinity, g/L
Water table and tide, m
Time, h

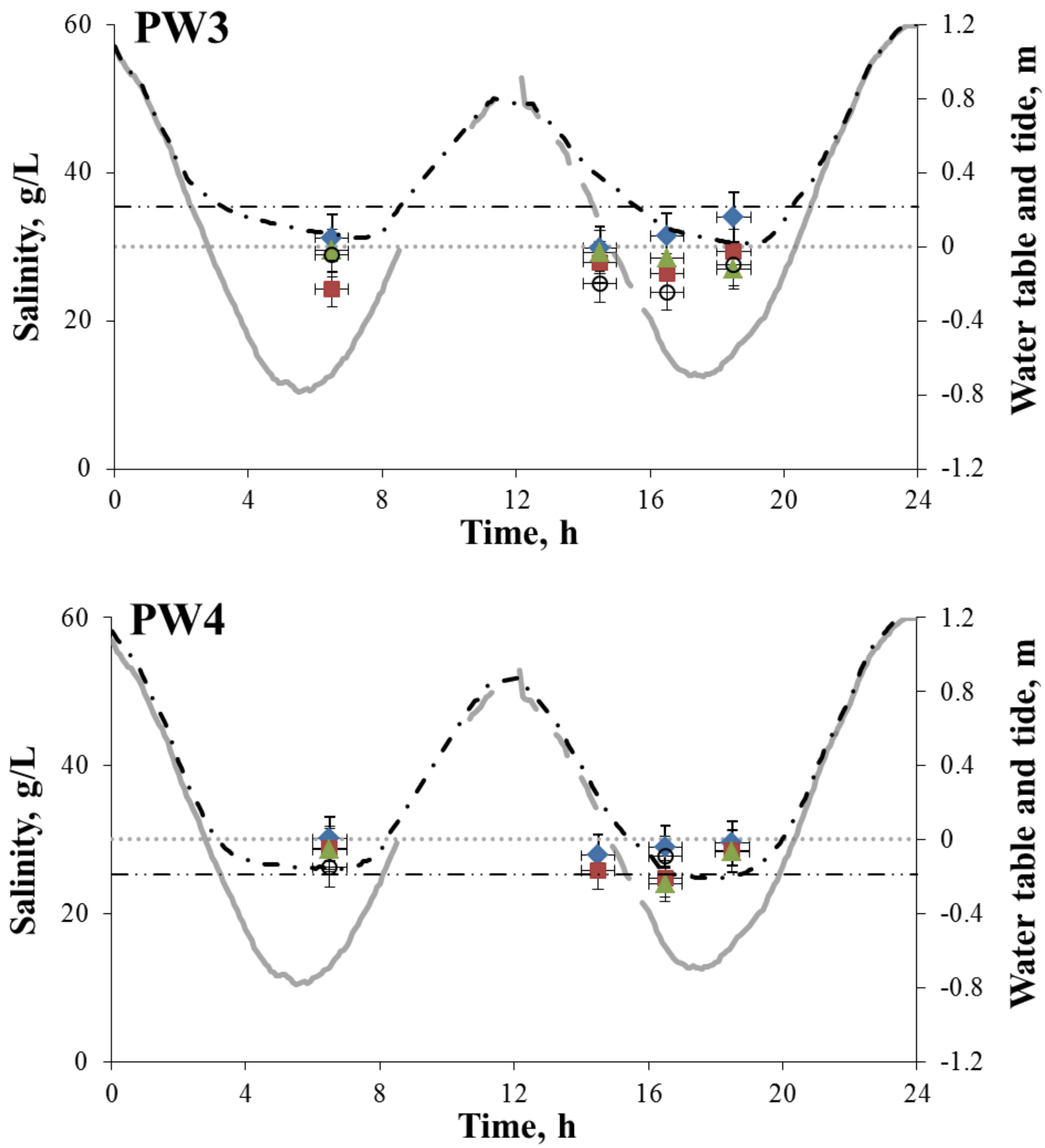

Supplementary Figure 5: Measured pore-water salinity during June 4th at four locations (PW1-PW4, Figure 1) and four depths, along with measurements of tide and groundwater table. Notice that high pore-water salinity was observed at the beach top layer at landward wells PW1 and PW2.

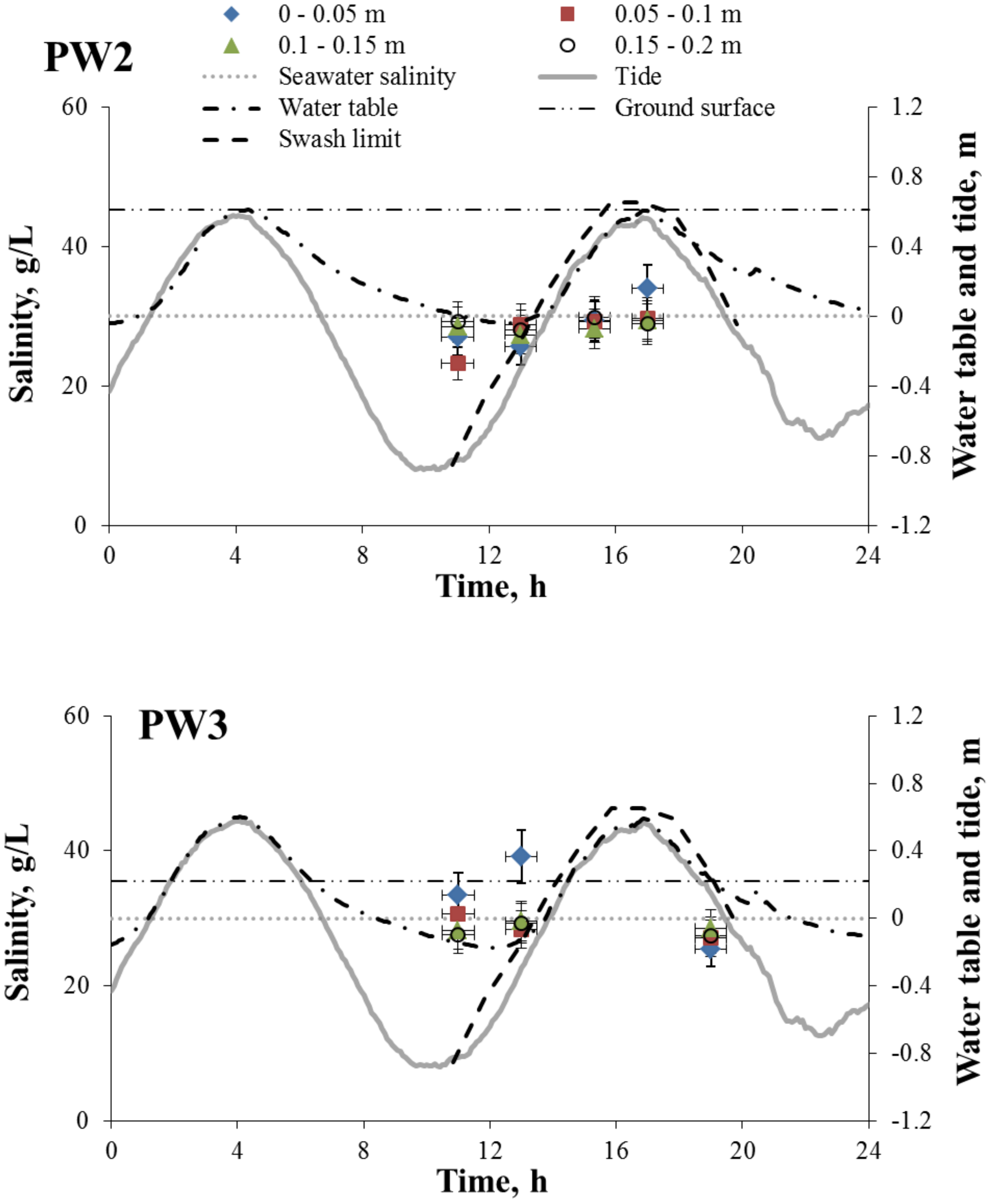
PW2
0 - 0.05 m
0.05 - 0.1 m
0.1 - 0.15 m
0.15 - 0.2 m
Seawater salinity
Tide
Water table
Ground surface
Swash limit
Salinity, g/L
Water table and tide, m
Time, h
PW3
Salinity, g/L
Water table and tide, m
Time, h

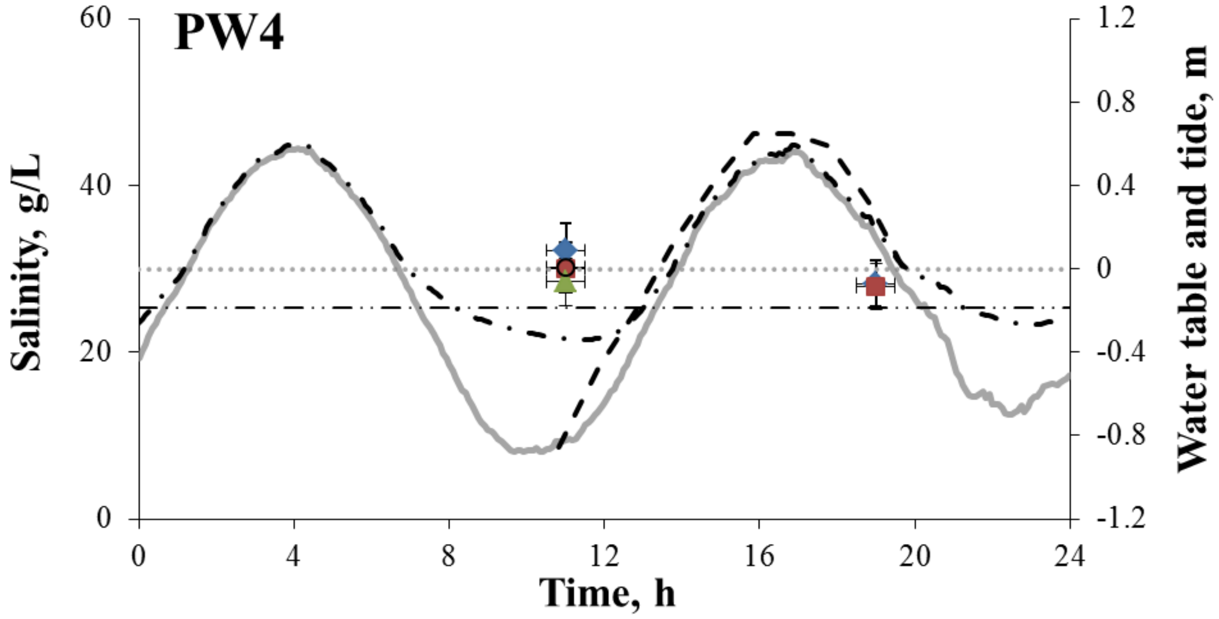

Supplementary Figure 6: Measured pore-water salinity during June 9th at four locations (PW2-PW4, Figure 1) and four depths, along with measurements of tide and groundwater table. Notice that the pore-water salinity was slightly higher at the beach top layer at PW wells.

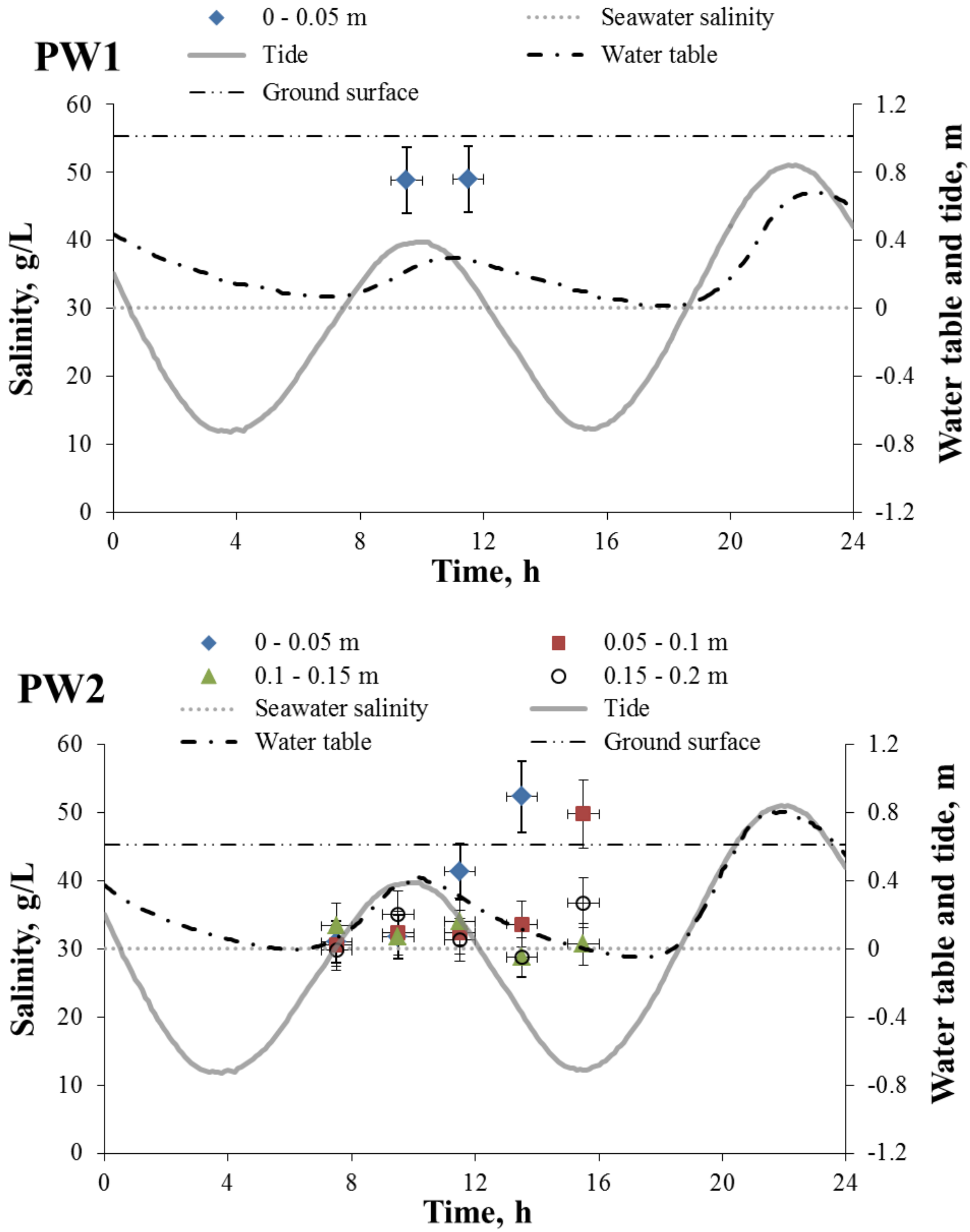
PW1
0 - 0.05 m
Seawater salinity
Tide
Water table
Ground surface
Salinity, g/L
Water table and tide, m
Time, h
PW2
0 - 0.05 m
0.05 - 0.1 m
0.1 - 0.15 m
0.15 - 0.2 m
Seawater salinity
Tide
Water table
Ground surface
Salinity, g/L
Water table and tide, m
Time, h

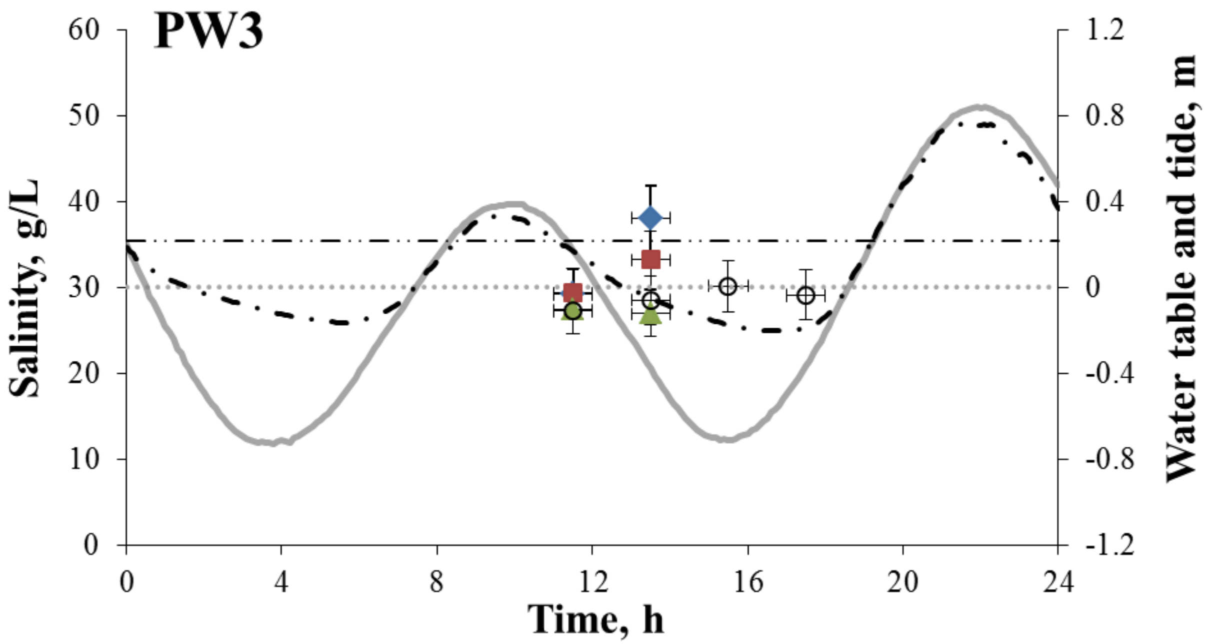

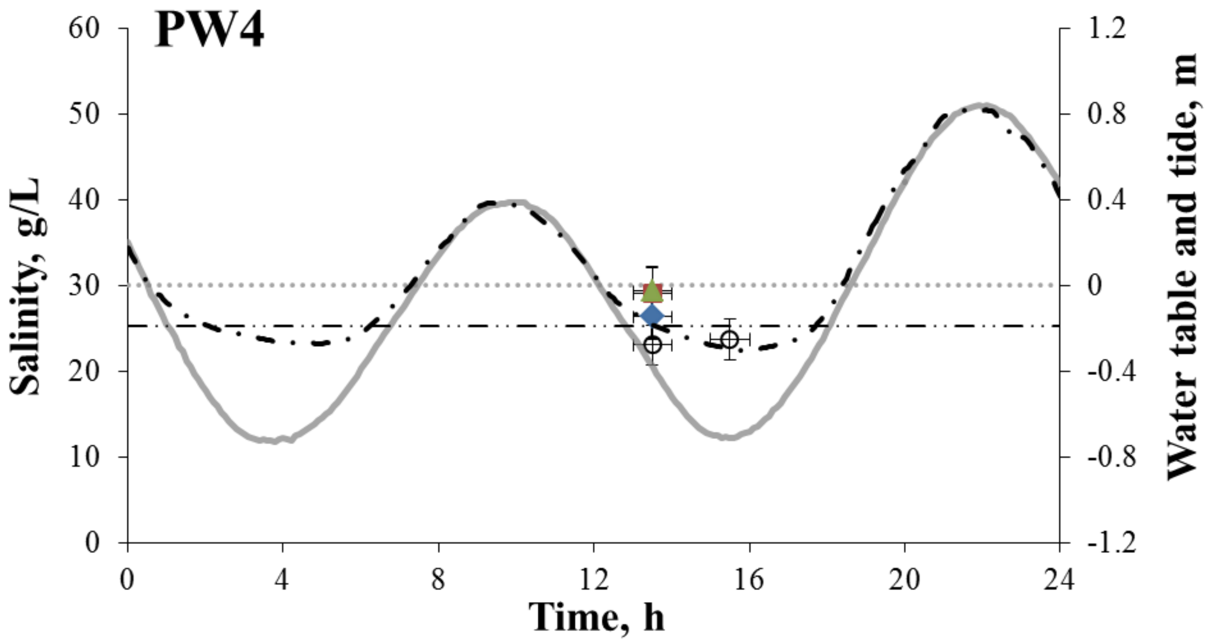

Supplementary Figure 7: Measured pore-water salinity during June 16th at four locations (PW1-PW4, Figure 1) and four depths, along with measurements of tide and groundwater table. Notice that high pore-water salinity was observed at the beach top layer at landward wells PW1 and PW2.

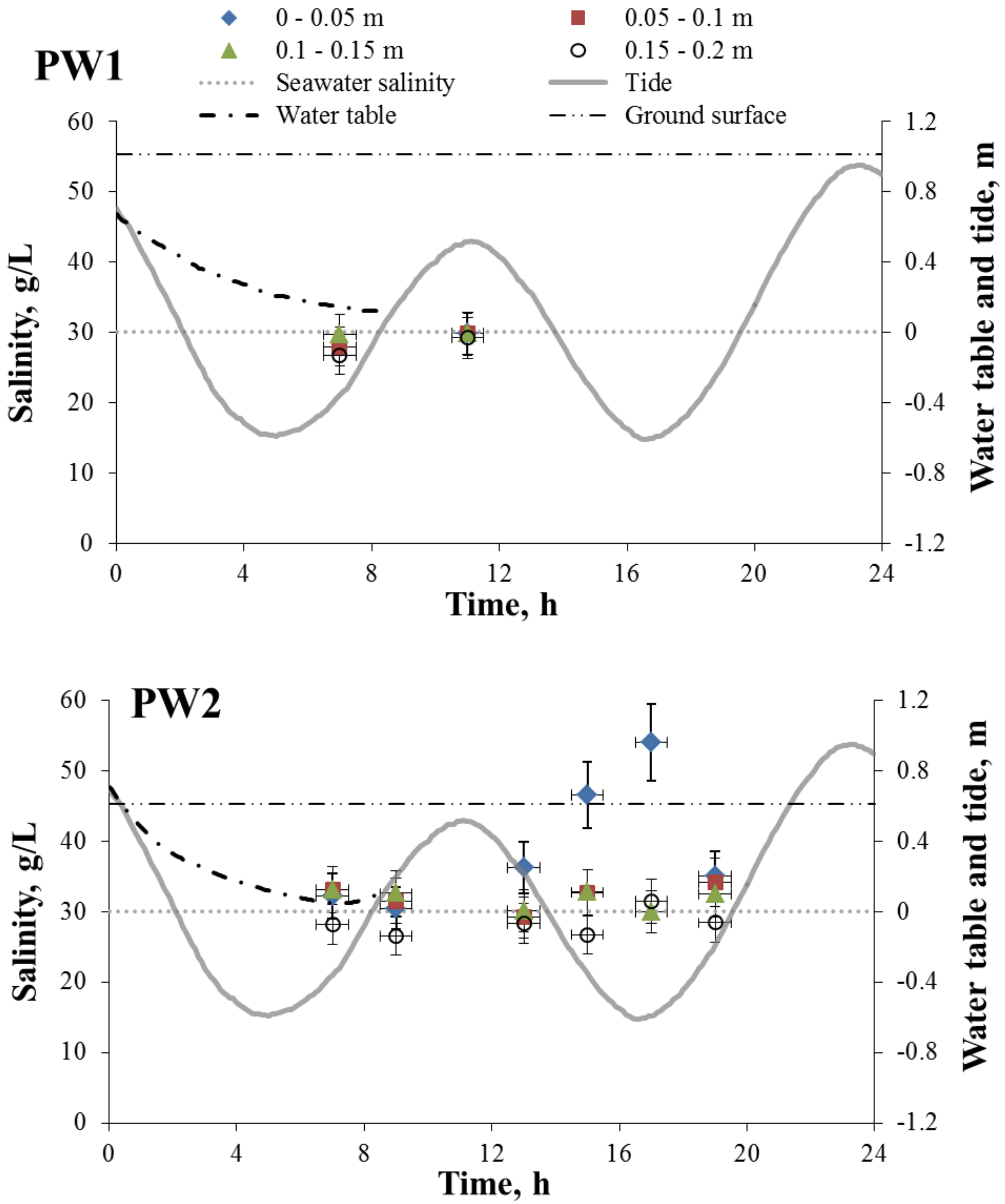

0 - 0.05 m
0.05 - 0.1 m
0.1 - 0.15 m
0.15 - 0.2 m
Seawater salinity
Tide
Water table
Ground surface
PW1
Salinity, g/L
Water table and tide, m
Time, h
PW2
Salinity, g/L
Water table and tide, m
Time, h

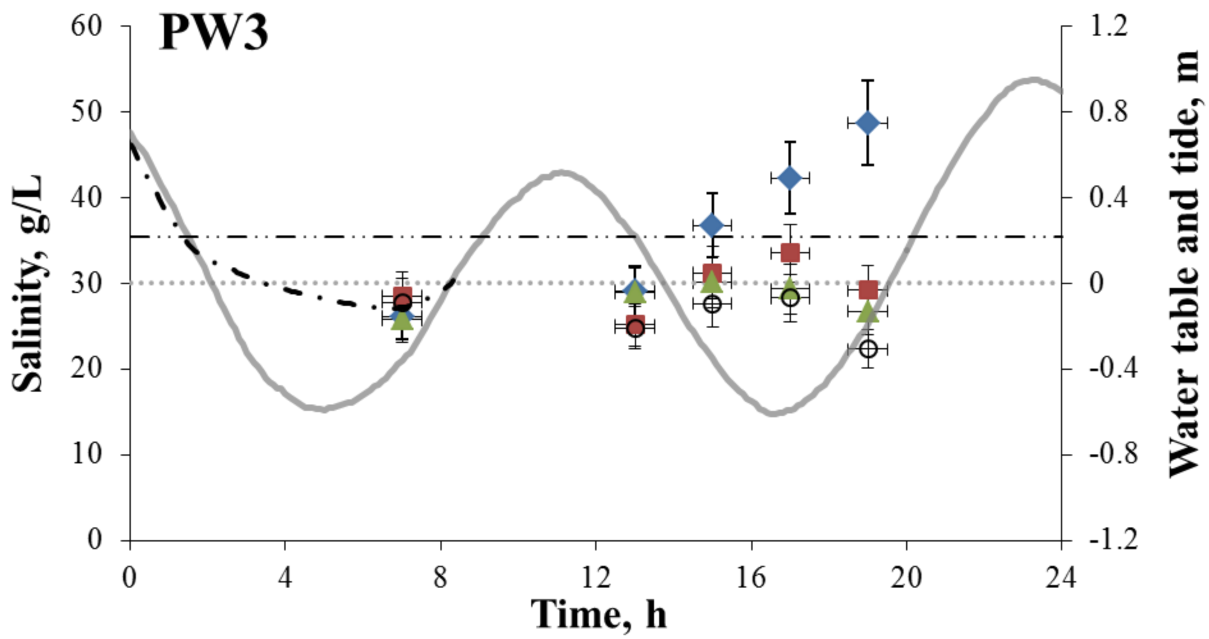

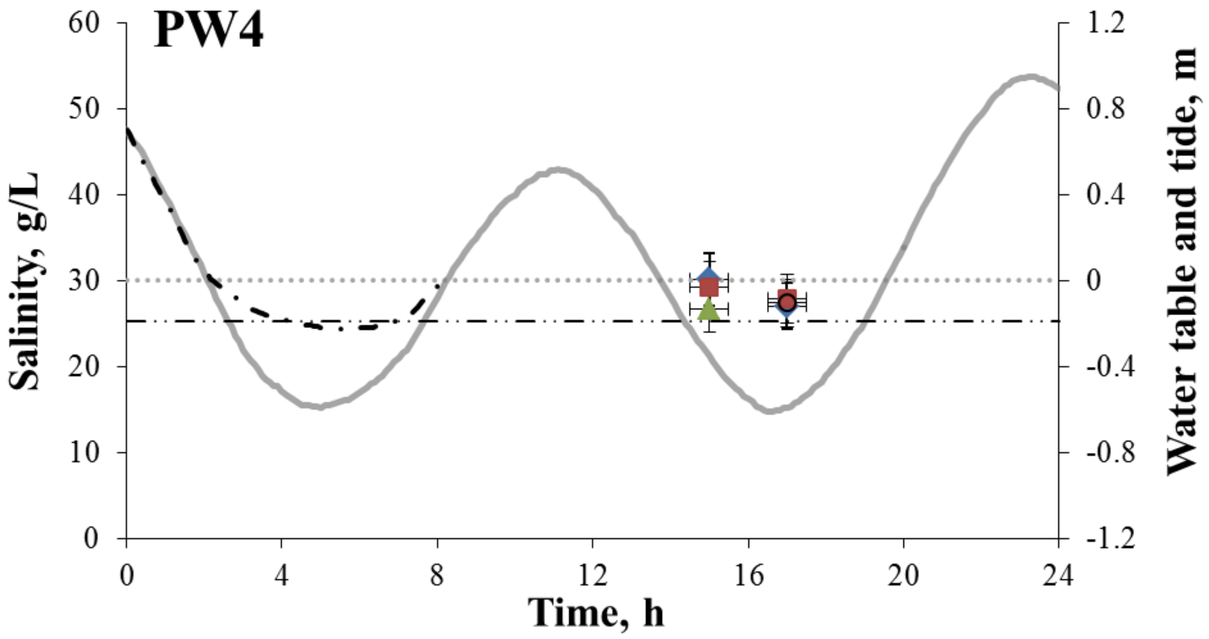

Supplementary Figure 8: Measured pore-water salinity during June 18th at four locations (PW1-PW4, Figure 1) and four depths, along with measurements of tide and groundwater table. Notice that high pore-water salinity was observed at the beach top layer at landward wells PW1 and PW2.

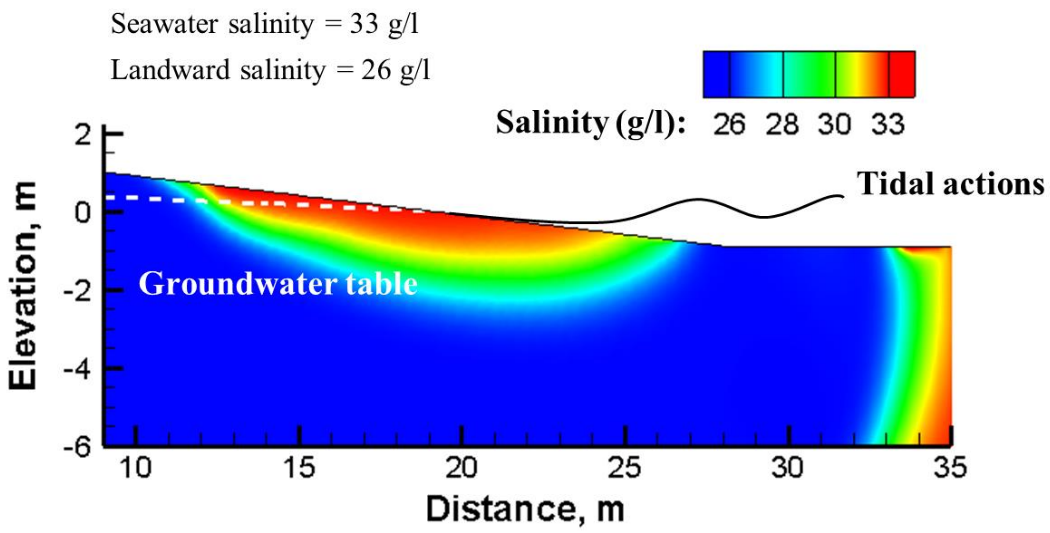

Supplementary Figure 9: Model schematic: initial concentration profile and dimensions. A mesh of 12,358 nodes (74 nodes in horizontal direction and 167 nodes in the vertical direction) was used to discretize the domain. It resulted in 24,236 triangular elements. A fine mesh resolution of Δz = 0.01 m was adopted at the top 0.3 m beach layer and Δz was increased from 0.02 m to Δz = 0.5 m at –6 m. The horizontal resolution of the mesh was Δx = 0.15 m between x = 9 m and x = 28 m, and Δx = 0.2 m between x = 28 m and x = 36 m. The mesh resolution is sufficiently fine to meet the strict criterion for the grid Péclet number to be less than or equal to 2.0[2,3]. The simulations were conducted for May 19th, based on observed data of tide, groundwater table, pore-water salinity, and meteorological conditions. The observed groundwater table in PW1 was used as landward boundary condition for the simulation. On the seaward side: when the beach surface was exposed to air, a Neumann boundary was assigned to the evaporation flux between ground surface and the atmosphere. For the submerged beach surface, the observed tide data was assigned as seaward boundary condition. A no-flow boundary condition was used for the bottom of the beach. Models were run in absence of evaporation until the hydraulic regime reached a quasi-steady state, and then the pressure and salinity distributions were used as initial conditions for the simulations taking into account evaporation effects. Variable time step, ranging from 0.1 s to 20.0 s, was used for the simulations to ensure that the grid Courant number was less than 1.0. In our study, the value of aerodynamic resistance was 50.0 s/m corresponding to the wind speeds of 2.0 m/s. The equation showing the relationship between aerodynamic resistance and wind speed is reported and discussed in Geng and Boufadel [4].

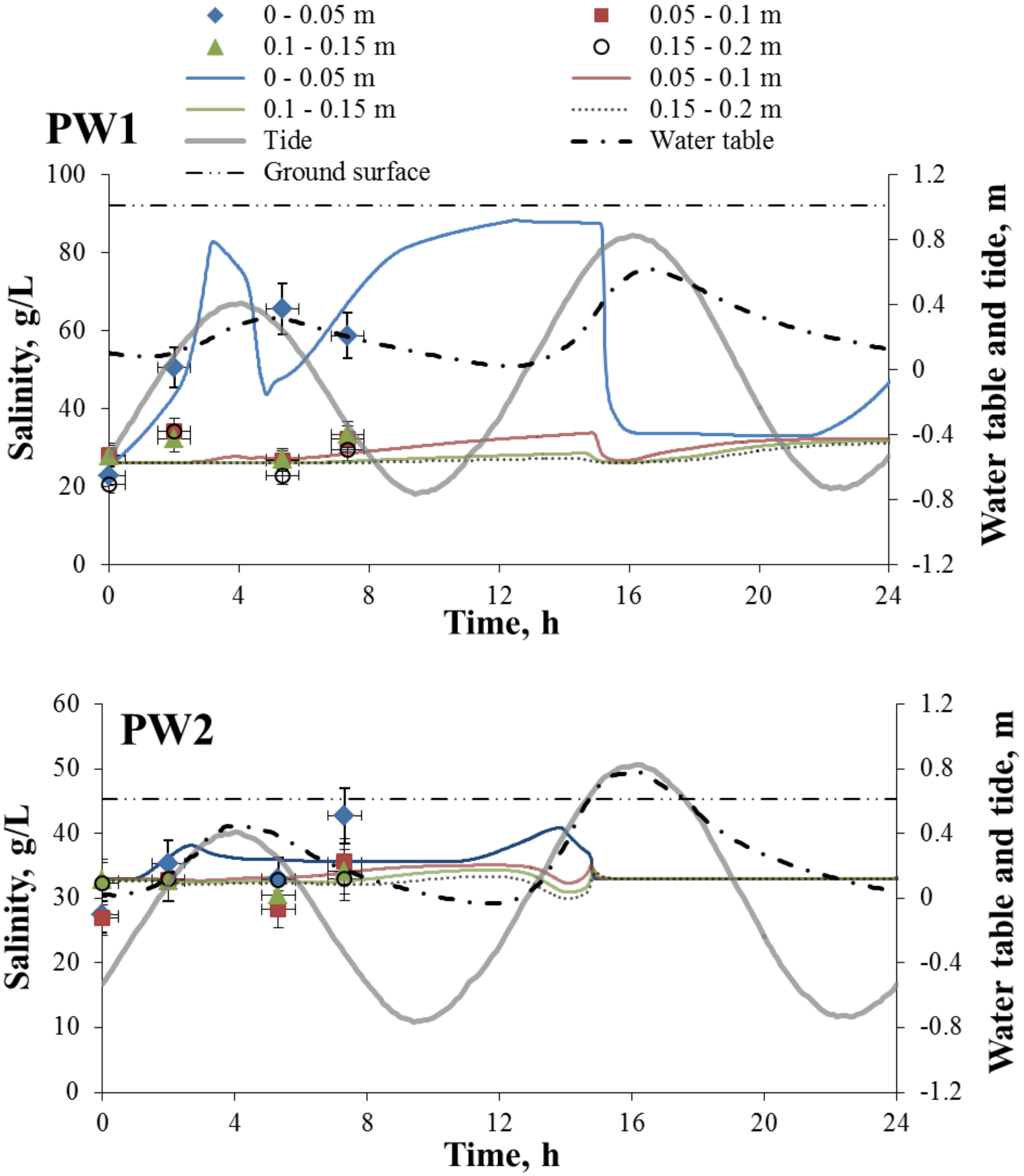
0 - 0.05 m
0.05 - 0.1 m
0.1 - 0.15 m
0.15 - 0.2 m
0 - 0.05 m
0.05 - 0.1 m
0.1 - 0.15 m
0.15 - 0.2 m
Tide
Water table
Ground surface
PW1
Salinity, g/L
Water table and tide, m
Time, h
PW2
Salinity, g/L
Water table and tide, m
Time, h

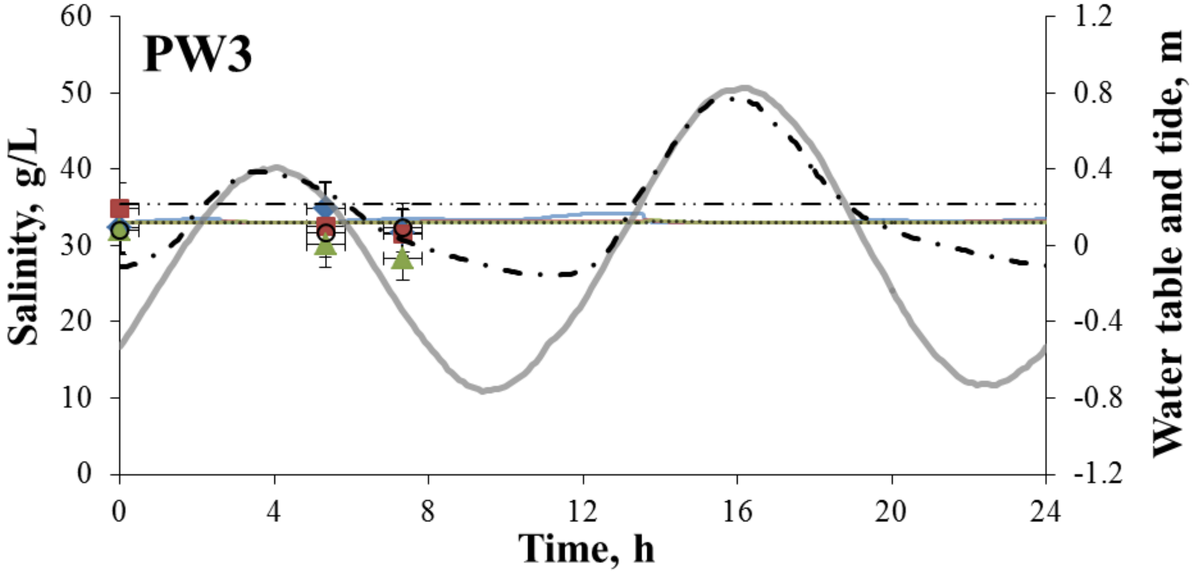

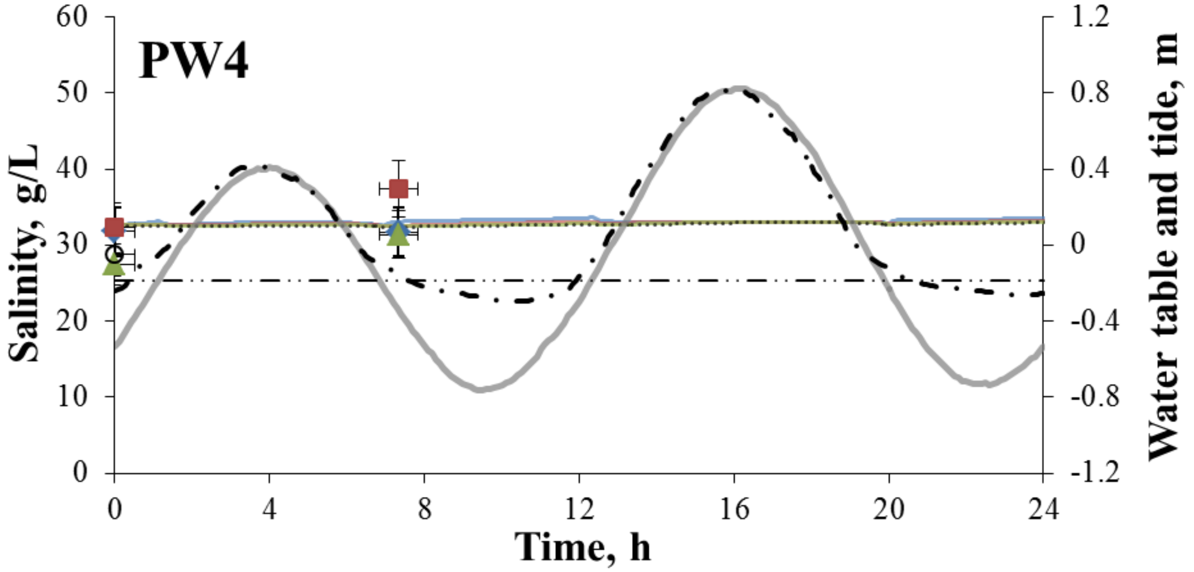

Supplementary Figure 10: Simulated (lines) and observed (symbols) pore-water salinity along with the measurements of tide and groundwater table during the observed period on May 19th, 2004. Time zero is midnight. Using one set of parameters (Table S1), the simulations captured closely the behavior of pore-water salinity variation observed at the PWs. The simulation results at PW1 show that the pore-water salinity at layer 0 – 0.05 m started to increase at t = 7.5 h, reaching a peak at t = 10.0 h after which the salinity decreased. After going through a "trough value" of 43.0 g/L at t = 12.0 h, the salinity increased again and reached 60 g/L at t = 14.5 h, in agreement with observations. While the increase in salinity was due to evaporation, the sudden decrease between t = 10.0 h and t = 12.0 h was due to the fact that high tide results in a shallow groundwater table which induces high moisture ratio near the surface. The latter would cause dilution of pore-water salinity near the beach surface. Compared to PW1, the pore-water salinity

at the shallow layer (0 – 0.05 m) of PW2 slightly increased from 30 g/L to 45 g/L during the observed period; the pore-water salinity almost remained a constant at the deeper location of PW1 and PW2, and at all depths of seaward wells PW3 and PW4 regardless of groundwater table fluctuation. This is probably due to a high moisture ratio there that facilitated vertical hydraulic connection and subsequently attenuated evaporation effects on pore-water salinity at the shallow layer of the beach. The results indicate a strong correlation between evaporation-induced high pore-water salinity near the beach surface (i.e., unsaturated zone) and coastal groundwater system (i.e., saturated zone).

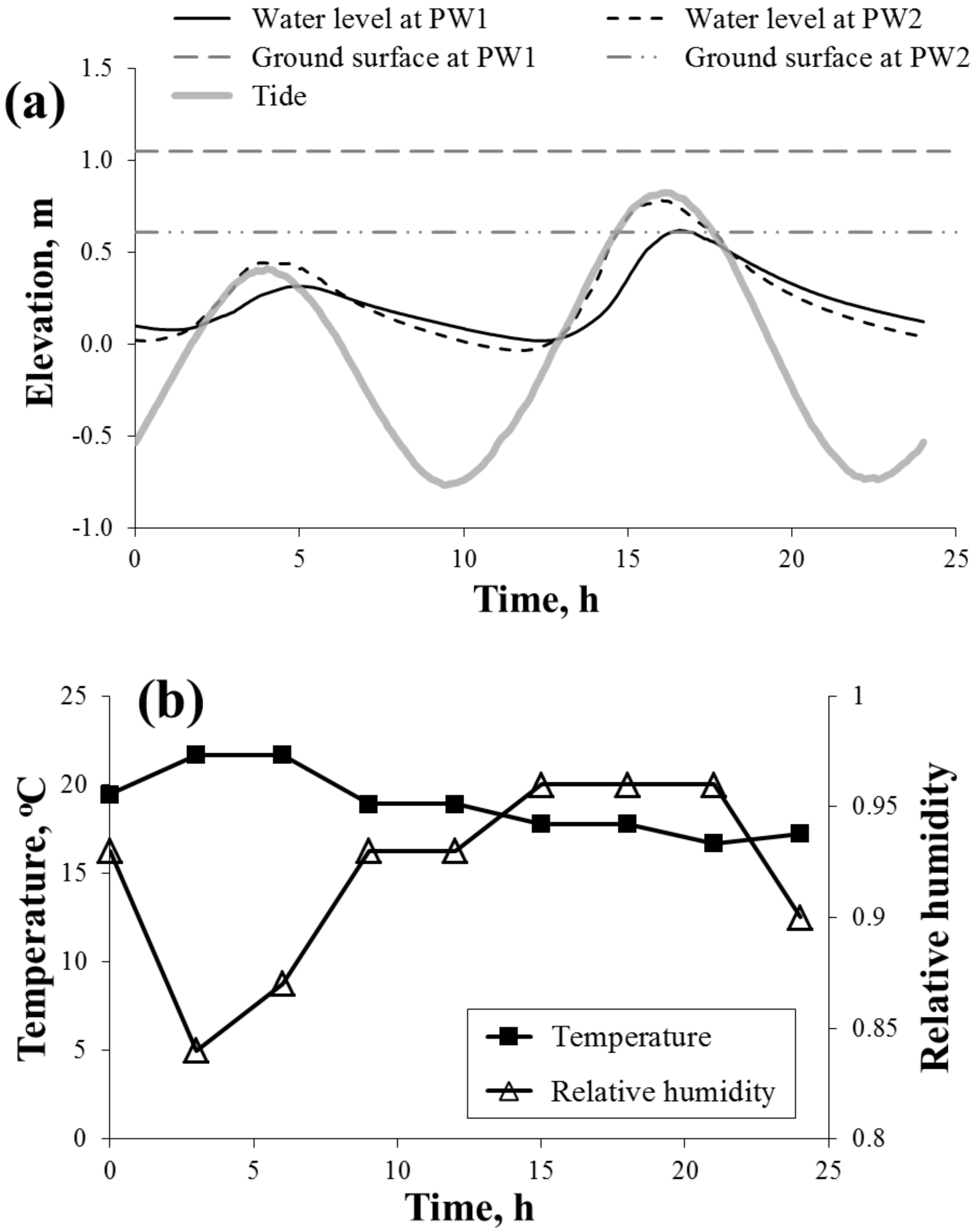

Supplementary Figure 11: measurements of (a) observed tide level and groundwater table and (b) air temperature and relative humidity during the period between 7:00 AM 19th May (i.e., t = 0 h) and 7:00 AM 20th May (i.e., t = 24 h). Numerical simulations (13 cases) were conducted during this period to further illustrate the model sensitivity to air temperature, relative humidity, and wind speed. Groundwater table and tide level were directly measured from the studied site. Data of air temperature and relative humidity were collected from NOAA, National Climatic Data Center. It is observed that air temperature varied between 17 ^{o}C and 22 ^{o}C during the day and the

relative humidity showed a large decrease at 10:00 AM. The lag time between tide peak and water table peaks increased moving landwards (i.e., from PW2 to PW1), which has been observed in prior studies[5].

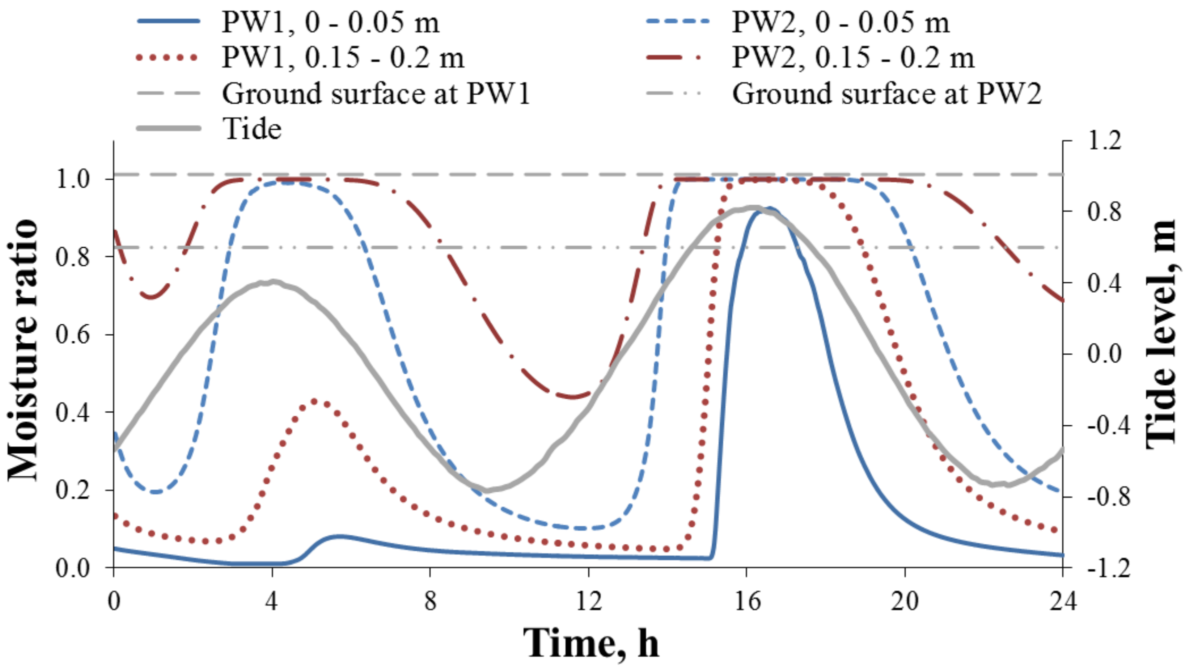

Supplementary Figure 12: Simulated moisture ratio at the shallow layers of (a) PW1 and PW2; (b) PW3 and PW4 during the observed period on May 19th. The simulated results shown in the Figure are depth averages of the layers 0 – 0.05 m and 0.15 – 0.2 m. The simulation results show that the moisture ratio at PW1 and PW2 decreased during the low tide and increased during the high tide. At PW1 location, relatively high moisture ratio at the layer of 0 – 0.05 m during the rising tide indicates dilution of pore water there which would attenuate evaporation-induced increase in pore-water salinity. This agrees with what was observed that pore-water salinity abruptly decreased when groundwater table was getting closer to the beach surface during the rising tide.

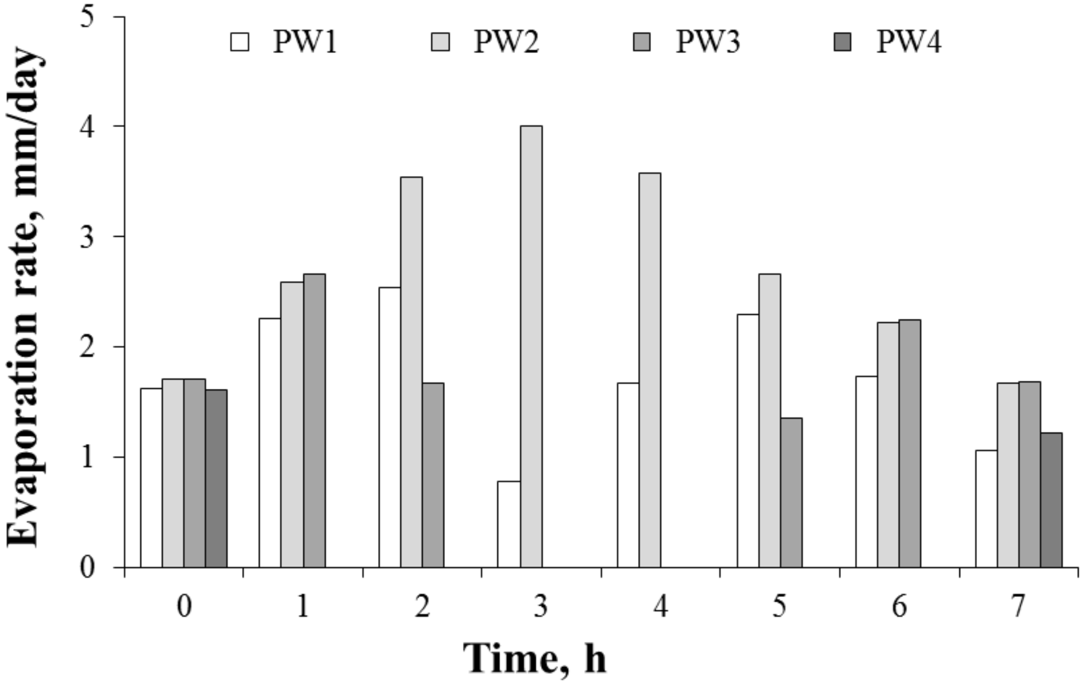

Supplementary Figure 13: Simulated evaporation rate at the well locations deployed along the transect during the observed period on May 19th. The simulation results show that at 10:00 AM, the evaporation rate at PW1 was very low (less than 1 mm/day), while that of PW2 reached maximum (i.e., 3.9 mm/day). Note that the relative humidity was the lowest at t = 10.0 h (Figure S11). At PW1 the soil moisture was near residual (i.e., 5%) while that at PW2 was equal to 15%, sufficiently large to cause high evaporation. The evaporation rate at PW3 and PW4 (during the beach exposure period) was on the average larger than those at PW1 and PW2. The results also show that the evaporation was relatively low at PW1 in comparison to other PWs, and thus the high salinity does not associate with a high evaporation flux, rather with a low soil moisture prior to evaporation occurring.

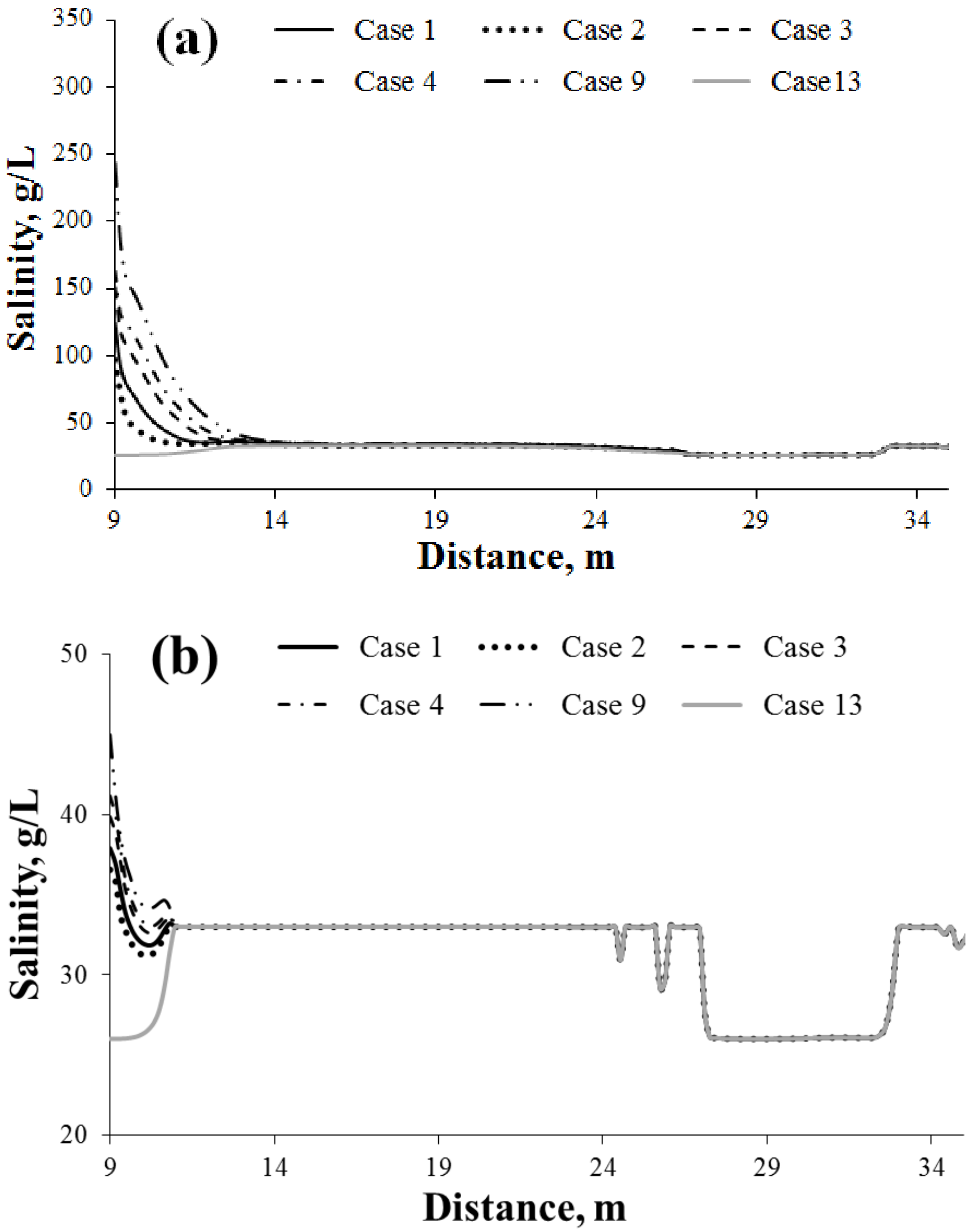

Supplementary Figure 14: Effect of temperature. Spatial distribution of pore-water salinity at top 5 cm layer along beach transect at (a) low tide (t = 9.5 h) and (b) high tide (t = 16.0 h) for Cases 1 – 4 using different temperature sets along with Case 9 (combined temperature and relative humidity effects) and Case 13 (without evaporation). The simulation results show that the pore-water salinity near the upper intertidal zone is very high during the low tide; and moving seawards, the pore-water salinity tended to decrease and leveled off at almost seawater salinity. This indicates that evaporation significantly impacted the pore-water salinity near the upper

intertidal zone; as moving seaward, the evaporation effects attenuated and the pore-water salinity was dominated by tide-induced seawater-groundwater recirculation. The salinity profile shows a concave between x = 27 m and x = 33 m. It is due to groundwater discharge. The results also indicate that the temperature effects on the pore-water salinity were greater during the low tide. At high tide, seawater infiltration from the surface narrowed the evaporation zone and diluted evaporation-induced high salinity; meanwhile, closer groundwater table to the beach surface increased surface moisture and diluted evaporation-induced high salinity zone from subsurface.

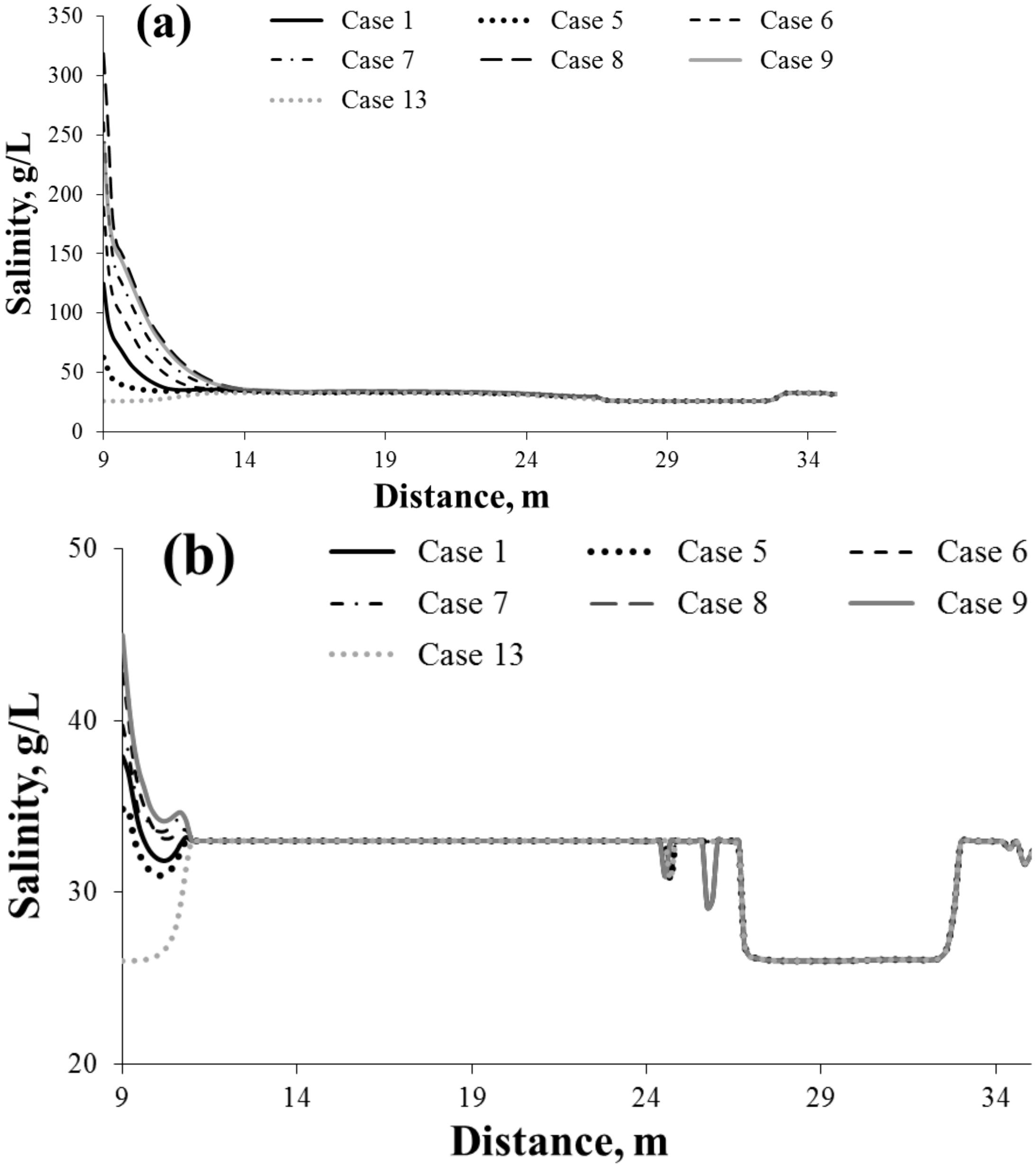

Supplementary Figure 15: Effect of relative humidity. Spatial distribution of pore-water salinity at top 5 cm layer along beach transect at (a) low tide (t = 9.5 h) and (b) high tide (t = 16.0 h) for Case 1 and Cases 5 – 8 using different relative humidity sets along with Case 9 (combined temperature and relative humidity effects) and Case 13 (without evaporation) for comparison. The simulation results show that besides increasing the pore-water salinity, lowering relative humidity also extended evaporation-induced high salinity zone more seawards. This spatial effect tended to be greater during the low tide.

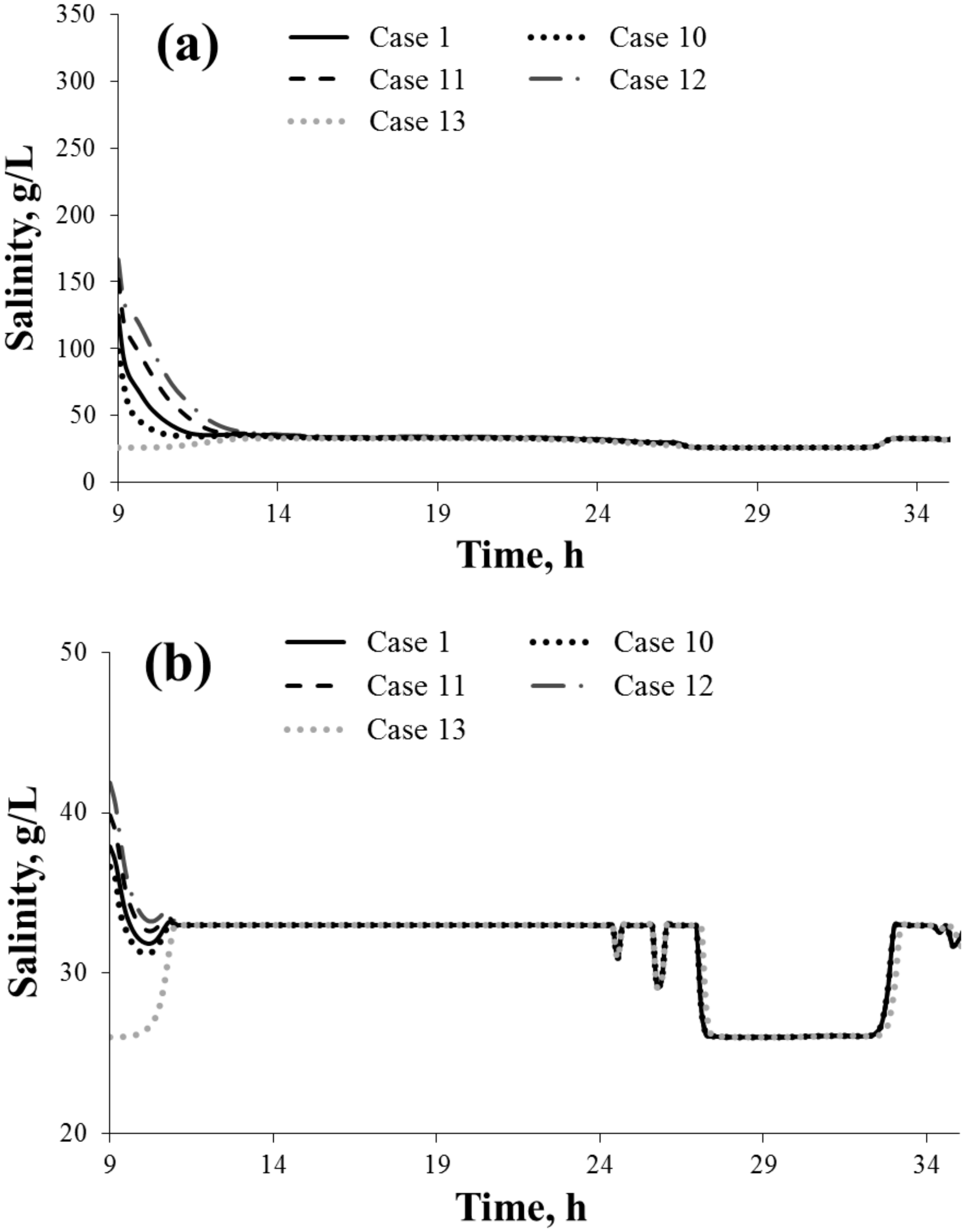

Supplementary Figure 16: Effect of wind speed. Spatial distribution of pore-water salinity at top 5 cm layer along beach transect at (a) low tide (t = 9.5 h) and (b) high tide (t = 16.0 h) for Case 1 and Cases 10 – 13 using different wind speed values along with Case 13 (without evaporation) for comparison. As expected, larger wind speed induced higher pore-water salinity near the upper intertidal zone and extended evaporation-induced high salinity zone more seawards.

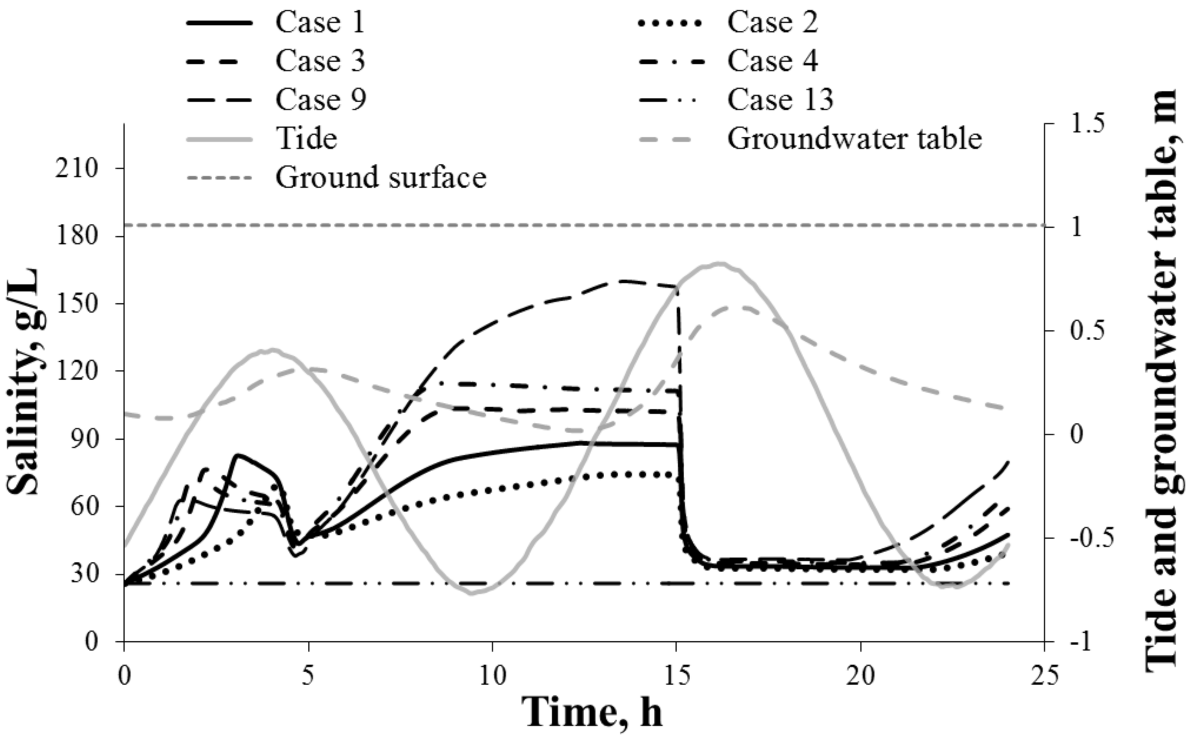

Supplementary Figure 17: Effect of air temperature. Temporal change of pore-water salinity at top 5 cm layer at the PW1 location (*x* = 9.0 m) for Cases 1 – 4 using different temperature sets along with Cases 9 and 13 for comparison. Notice that the relative humidity sets used in Cases 2 – 4 are the same as that of Case 1, while the relative humidity set used in Case 9 is the same as that of Case 6. Case 13 was conducted without evaporation. The simulation results show that rather than simply increasing pore-water salinity, temperature change modified the temporal response of the pore-water salinity to beach evaporation. It is because that higher temperature caused higher evaporation on the beach surface and thereby resulted in faster extraction of pore water from the beach surface.

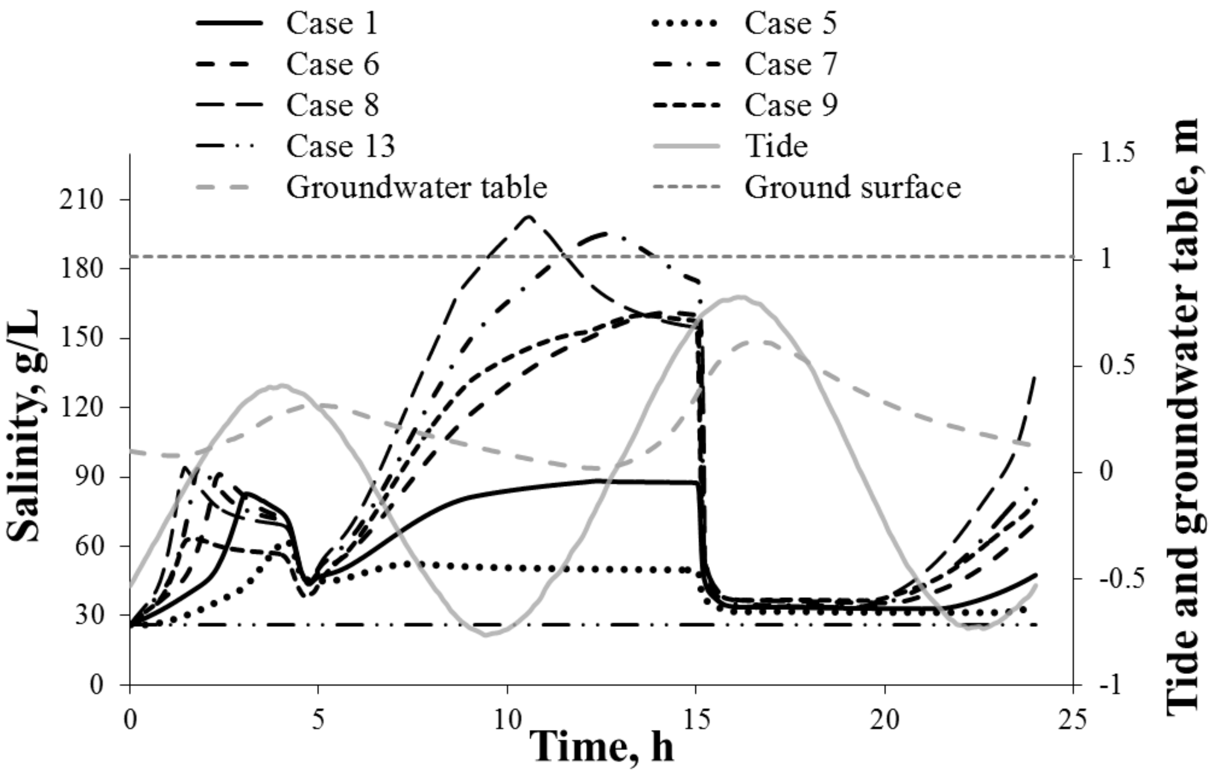

Supplementary Figure 18: Effect of relative humidity. Temporal change of pore-water salinity at top 5 cm layer at the PW1 location (x = 9.0 m) for Cases 1 and 5 – 8 using different relative humidity sets along with Cases 9 and 13 for comparison. Notice that the temperature sets used in Cases 5 – 8 are the same as that of Case 1, while the temperature set used in Case 9 is the same as that of Case 3. Case 13 was conducted without evaporation. The simulation results show that change of relative humidity in the air modified temporal response of pore-water salinity at shallow layer of the beach and lower relative humidity induced faster response of pore-water salinity to the evaporation. The results also show that superimposing the effects of air temperature and relative humidity further intensified the increase in the pore-water salinity at shallow layer of the beach.

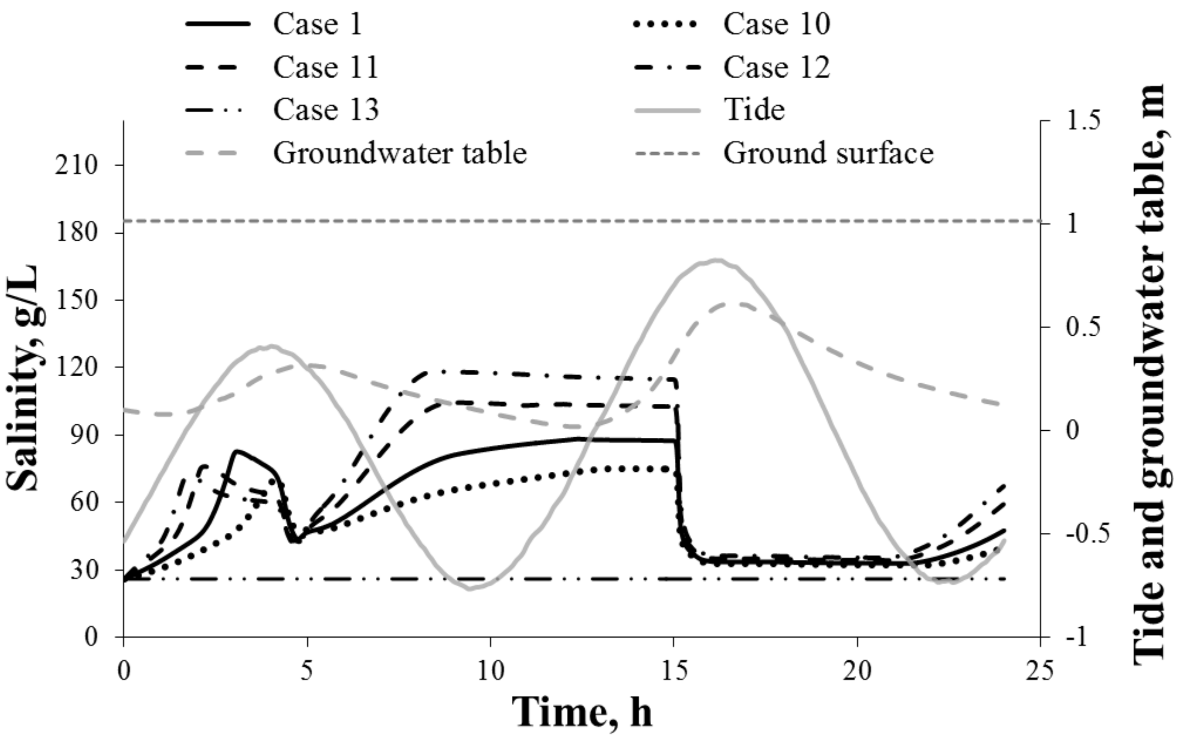

Supplementary Figure 19: Effect of wind speed. Temporal change of pore-water salinity at top 5 cm layer at the PW1 location (x = 9.0 m) for Cases 1 and 10 – 12 using different wind speed along with Case 13 (without evaporation) for comparison. The simulation results show that change of wind speed modified temporal response of pore-water salinity at shallow layer of the beach and higher wind speed induced faster response of pore-water salinity to the evaporation.

Reference

1 Bobo, A. M., Khoury, N., Li, H. & Boufadel, M. C. Groundwater Flow in a Tidally Influenced Gravel Beach in Prince William Sound, Alaska. *Journal of Hydrologic Engineering* **17**, 494-494 (2012).

2 Zheng, C. & Bennett, G. D. Applied contaminant transport modeling. *Industrial and Commercial Training* **34**, 256-262 (2002).

3 Boufadel, M. C., Suidan, M. T. & Venosa, A. D. Density-dependent flow in one-dimensional variably-saturated media. *Journal of Hydrology* **202**, 280-301 (1997).

4 Geng, X. & Boufadel, M. C. Impacts of evaporation on subsurface flow and salt accumulation in a tidally influenced beach. *Water Resources Research* **51**, 5547-5565 (2015).

5 Nielsen, P. Tidal dynamics of the water table in beaches. *water resources research* **26**, 2127-2134 (1990).
